# Trajectories of self-reported fatigue following initiation of multiple sclerosis disease-modifying therapy

**DOI:** 10.1136/jnnp-2024-333595

**Published:** 2024-05-14

**Authors:** Simon Englund, Thomas Frisell, Ying Qu, Kavita Gandhi, Annika Hultén, Marie Kierkegaard, Fredrik Piehl, Elisa Longinetti

**Affiliations:** 1 Department of Clinical Neuroscience, Karolinska Institutet, Stockholm, Sweden; 2 Clinical Epidemiology Division, Department of Medicine Solna, Karolinska Institutet, Stockholm, Sweden; 3 GCSO, Janssen Pharmaceuticals, Stockholm, Sweden; 4 Janssen Research & Development, LLC, Titusville, New Jersey, USA; 5 Janssen-Cilag Oy, Espoo, Finland; 6 Department of Neurobiology, Care Sciences and Society, Karolinska Institutet, Stockholm, Sweden; 7 Academic Specialist Center, Center of Neurology, Stockholm Health Services, Stockholm, Sweden

**Keywords:** clinical neurology, multiple sclerosis, epidemiology

## Abstract

**Background:**

We analysed the COMparison Between All immunoTherapies for Multiple Sclerosis (NCT03193866), a Swedish nationwide observational study in relapsing–remitting multiple sclerosis (RRMS), to identify trajectories of fatigue and their association with physical disability following start of disease-modifying therapy (DMT).

**Methods:**

Using a group-modelling approach, we assessed trajectories of fatigue with the Fatigue Scale for Motor and Cognitive Functions and physical disability with Expanded Disability Status Scale among 1587 and 1818 individuals who initiated a first DMT and had a first DMT switch, respectively, followed during 2011–2022. We investigated predictors of fatigue trajectories using group membership as a multinomial outcome and calculated conditional probabilities linking membership across the trajectories.

**Results:**

We identified five trajectories of fatigue in participants who initiated their first DMT: no fatigue (mean starting values=23.7; 18.2% of population), low (35.5; 23.9%), mild (49.0; 21.6%), moderate (61.3; 20.1%) and severe (78.7; 16.1%). While no, low, mild and severe fatigue trajectories remained stable, the moderate trajectory increased to severe fatigue. Similarly, we identified six fatigue trajectories among participants who did a DMT switch, all indicating stable values over time. Women initiating a first DMT were more likely than men to display a severe fatigue trajectory, relative to the no fatigue one. There was a strong association between fatigue and physical disability trajectories.

**Conclusions:**

In this cohort of people with actively treated RRMS, self-reported fatigue remained stable or increased over the years following DMT start. There was a strong association between fatigue and disability after DMT start.

**WHAT IS ALREADY KNOWN ON THIS TOPIC**

- Fatigue is recognised as one of the most prevalent and debilitating symptoms of multiple sclerosis (MS). However, the longitudinal trajectory of fatigue in patients with relapsing–remitting multiple sclerosis (RRMS) following the initiation of disease-modifying therapy (DMT) and first DMT switch in contemporary therapeutic settings remains unclear.

**WHAT THIS STUDY ADDS**

- In this population-based cohort of people with actively monitored RRMS from first DMT start (n=1587) and from first DMT switch (n=1818), those with no, low, mild or severe fatigue at first DMT start maintained stable fatigue ratings, whereas those with moderate fatigue experienced further deterioration. Fatigue remained stable after second ever therapy initiation, regardless of initial fatigue rating.

**HOW THIS STUDY MIGHT AFFECT RESEARCH, PRACTICE OR POLICY**

- Individuals who exhibit moderate fatigue at first start of a DMT may represent a high-risk group susceptible to further fatigue deterioration.

## Introduction

 Self-reported fatigue, a prevalent and debilitating symptom affecting up to two-thirds of people with multiple sclerosis (PwMS),^1^ strongly correlates with quality of life (QoL).^2^ The cause of fatigue in MS is unknown but seems to involve a combination of disease-related mechanisms and non-disease-specific factors.^3^ Understanding these mechanisms, predictors and associations is important for developing management strategies and improving QoL. Evidence points to inflammation within the central nervous system, diffuse demyelination, axonal lesions and brain atrophy as potential contributors to fatigue,^3^ alongside non-disease-specific factors like sleep disturbances, heat and depression.^3 4^

Studies on over time fatigue changes in PwMS are contradictory. While some indicate a decrease,^5^,^7^ others report increasing,^8^ stable^9^,^13^ or fluctuating fatigue ratings.^9 14^ Predictors of fatigue changes also vary; some studies identify depression, pain and baseline fatigue,^914^,^17^ while covariates like age, sex, Expanded Disability Status Scale (EDSS) and lifestyle factors inconsistently predict fatigue.^914^,^18^ Research leveraging MRI indicate that evolving brain atrophy in specific brain regions contribute both to disability progression^19^ and worsening of fatigue.^20^ However, epidemiological studies found no evident link between changes in fatigue and evolution of physical disability.^6 10^ Moreover, there is limited information on whether effective disease-modifying therapies (DMTs) impact fatigue trajectories.^21^

Large, high-quality, long-term population-based studies are needed to understand the extended course of fatigue in MS, its relationship with physical disability and the potential benefits of switching to a more effective DMT. To fill these knowledge gaps, we followed 1587 and 1818 study participants with relapsing–remitting multiple sclerosis (RRMS) over 11 years, starting from the initiation of a first ever DMT (‘*first DMT’*) and after switching DMT (‘*DMT switch’*), respectively, linking data from the Swedish nationwide observational study in RRMS, COMparison Between All immunoTherapies for Multiple Sclerosis (COMBAT-MS), to several Swedish national registers. Our objectives were to (1) identify fatigue trajectories and their associations with physical disability trajectories, (2) explore predictors of fatigue trajectory groups and (3) evaluate the impact of DMT switch on fatigue trajectories.

## Methods

In a population-based cohort study of persons with RRMS, we assessed fatigue and physical disability trajectories linking COMBAT-MS data to the Swedish MS Registry and national healthcare and census registers. The study was approved by the Swedish Ethical Review Authority (202102384).

### Study design

The COMBAT-MS (NCT03193866) is a prospective population-based non-intervention drug study in RRMS. Out of an eligible study population of about 3800 (~50% of the nationwide prevalence), the COMBAT-MS cohort included 3397 PwMS who started a first DMT or did a first DMT switch between 2011 and 2018 at any of the 10 MS clinics affiliated with Sweden’s seven medical university faculties. Prospective data from annual assessments from 2017 to 2022 were entered into the Swedish MS Registry,^22^ an integrated web-based part of Swedish MS care collecting results of disability ratings and MRI, information on DMT use and various patient-reported outcomes, including Fatigue Scale for Motor and Cognitive Function (FSMC).^23^ Data from 2011 to 2017 were retrieved retrospectively along with a medical chart review to ensure validity.^23^

### Study population

**Figure 1:**
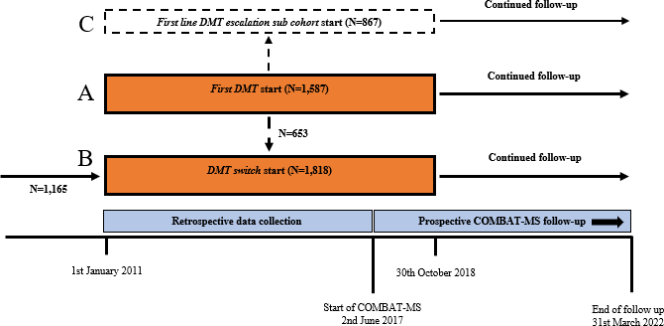
Schematic illustration of 2752 unique study participants who initiated their *first DMT* (**A**) or had a first *DMT switch* (**B**) between 2011 and 2018. A dotted line connects *first DMT* cohort (**A**) and *DMT switch* cohort (**B**), indicating that 653 participants fulfilled the inclusion criteria for both cohorts and contributed once to each cohort with different treatment episodes. The solid line connects to cohort B, representing the 1165 participants in this cohort who initiated their first DMT before 2011. *First line DMT escalation subcohort* (**C**) is a subset of the first DMT cohort (**A**). Data were collected prospectively from annual assessments between 2017 and 2022 and retrieved retrospectively along with a medical chart review between 2011 and 2017. COMBAT-MS, COMparison Between All immunoTherapies for Multiple Sclerosis; DMT, disease-modifying therapy.

Figure 1 shows inclusion and monitoring timelines. Our study population included all participants of the COMBAT-MS study (RRMS; age 18–75 at inclusion; starting a first DMT or doing a first DMT switch between 2011 and 2018; NCT03193866 for further inclusion and exclusion criteria). Additional study-specific criteria applied here were to have received either dimethyl fumarate, fingolimod, glatiramer acetate, interferons (interferon beta-1a, peginterferon beta-1a and interferon beta-1b), natalizumab, rituximab or teriflunomide as *first DMT*, or dimethyl fumarate, fingolimod, natalizumab, rituximab or teriflunomide as *DMT switch*, to be residing in Sweden from ≥5 years prior to MS diagnosis, and to have at least three FSMC^24^ total scores recorded after DMT start (n=1587 in *first DMT*, n=1818 in *DMT switch*). We cross-linked our study population through a unique personal identification number to the Total Population Register,^25^ and followed participants from DMT start until emigration, death, withdrawal of consent or end of follow-up (12 May 2022), whichever came first.

### Fatigue and physical disability assessment

Fatigue and physical disability were assessed via annual assessment of patient completed FSMC total^24^ and physician completed EDSS scores during the conduct of COMBAT-MS, with additional FSMC total and EDSS scores recorded as part of routine care.

### Predictors of fatigue trajectories

Potential predictors of fatigue trajectories were probed at DMT start by cross-linking data to the following demographic and health registries: Population,^26^ Patient,^27^ Prescribed Drug,^28^ Swedish social insurance agency and the longitudinal database for insurance and labour market studies.^29^ Information collected included age, sex, region of residence, country of birth, educational level, days on sick leave and disability pension, prevalence of specific comorbid conditions, prescribed treatment dispensation, MS disease-related characteristics (DMT, MS duration, recent relapse, recent accrual of new T2 lesions on brain MRI), and scores of FSMC, EDSS, Symbol Digit Modalities Test, the physical and psychological components of the MS Impact Scale^30^ (MSIS-29), and EuroQol-visual analogue scale (EQ-5D VAS) score and MS-symptoms inventory (a modification of the Guy’s Neurological Disability Scale,^31^ see online supplemental eTable 1 for details).

### Statistical analysis

Applying a similar strategy as in a previous study,^32^ we identified distinctive clusters of individual FSMC total and EDSS trajectories using group-based trajectory modelling.^33^

In censored normal models, we linked time since DMT start and FSMC total and EDSS scores through a polynomial relationship. We conducted model selection using the Bayesian information criteria.^33^ First, we determined the optimal number of trajectory groups with a quadratic form and 10% minimum group size requirement. Then, we identified the polynomial function order for each group’s trajectory, specifying the shape of each group up to a cubic function. In the *first DMT* cohort, the best fitting models had five fatigue trajectories (two linear and three quadratic functions of time since DMT start), and three for physical disability (a cubic, a linear and a quadratic function). In the *DMT switch* cohort, six fatigue trajectories (all linear functions) and three for physical disability (a cubic and two linear functions) best fit the data. Finally, participants were assigned to the trajectory with the highest posterior membership probability for each individual. The models were adequate, with average posterior probability (AvePP) above the ≥0.70 recommended criteria^33^ (*first DMT* cohort: fatigue trajectories 0.90–0.95, physical disability trajectories 0.96–0.97; *DMT switch* cohort: fatigue trajectories 0.88–0.94, all physical disability trajectories 0.97).

To analyse associations between dynamic evolution of FSMC total and EDSS scores as two distinct but related outcomes, we used joint trajectory models^34^ and reported conditional probabilities linking membership across the trajectory groups of the two scales.

To understand how individual characteristics impact FSMC total trajectories, we analysed potential predictors (listed above) by using group membership assignments as a multinomial outcome. ORs and corresponding 95% CIs were derived using multinomial logistic regression models. Each predictor was first analysed separately and then progressively inserted in the full model.

For the main analysis, we applied an intention-to-treat approach. To further evaluate the effect of a DMT switch on fatigue trajectories, we defined a subcohort within the *first DMT* cohort, referred to as the ‘first line DMT escalation’ (figure 1). This subcohort includes those who switched from a first-line DMT (dimethyl fumarate, glatiramer acetate, interferons or teriflunomide) to an escalation DMT (alemtuzumab, cladribine, fingolimod, natalizumab, ocrelizumab, rituximab, Autologous haematopoietic stem cell transplantation, siponimod or daclizumab), as well as those who did not switch to an escalation DMT. We incorporated DMT switch as a time-varying covariate at 6-month intervals from first DMT start, to compare fatigue trajectories between those who did not switch to an escalation DMT and those who switched to an escalation DMT at these specific time points. To investigate differential effects of the DMT switch by fatigue trajectory group, we performed Wald test on model parameter estimates.

### Missing data

Information at DMT start was missing for <1% of potential predictors of fatigue trajectories, excluding MS disease-related characteristics (missingness 11%–87%, online supplemental eTable 2). We addressed missing baseline covariate values with multiple imputation using chained equations. Each missing variable was imputed as a function of all other baseline variables used in the analysis (plus their transformations), and the predicted trajectory group membership probabilities. Predictive mean matching was used for quantitative variables and logistic regression for categorical variables, with models defined using fully conditional specification, 20 imputations and 10 burn-in iterations. Confidence intervals around group contrasts was constructed by pooling effect estimates and variance using Rubin’s rules.^35^

### Sensitivity analyses

To investigate differences across fatigue subdomains, we repeated analyses for FSMC cognitive and motor scores separately. To address trajectory misclassification, we investigated predictors of trajectories after excluding participants with trajectory assignment probabilities <0.80.

All analyses were made with Stata V.16.1 and SAS V.9.4.

### Data sharing statement

Data may be obtained from a third party and are not publicly available.

## Results

Between 2011 and 2022, 1587 participants were followed for an average of 7.1 years (2.2 SD) from *first DMT* start, and 1818 participants were followed for an average of 7.3 years (1.9 SD) from *DMT switch* start, with mean FSMC total scores collected per person of 5.4 (2.1 SD) and 5.7 (2.5 SD), respectively. The average time between MS diagnosis and *first* DMT start was 1.0 year (3.3 SD) and between MS diagnosis and *DMT switch* start was 6.3 years (5.8 SD). Due to death or emigration, 21 participants were lost to follow-up.

### Fatigue trajectories

**Figure 2:**
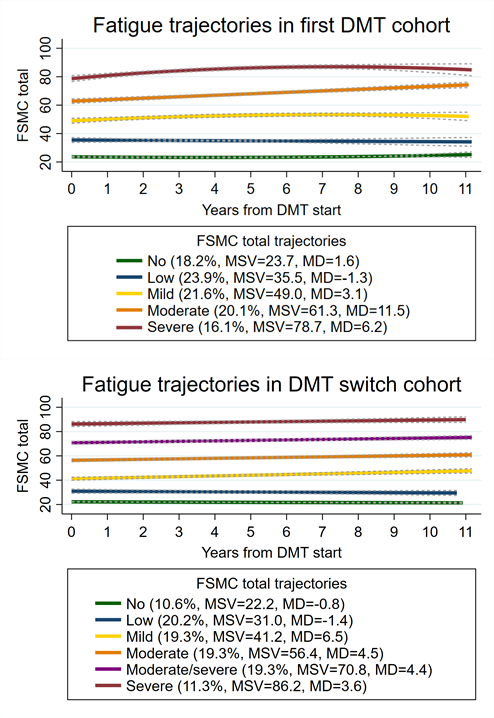
Trajectories of fatigue (FSMC) and corresponding 95% CIs (dotted lines), over 11 years following first (upper panel) and switch (lower panel) DMT initiation. Trajectory group legends describe the proportion of participants in each group relative to the entire study population, mean FSMC total starting values and the difference in FSMC total values compared with baseline. DMT, disease-modifying therapy; FSMC, Fatigue Scale for Motor and Cognitive function; MD, Mean Difference; MSV, mean starting value.

Figure 2 displays estimated mean FSMC total scores in fatigue trajectories at each year post-DMT start over 11 years for the *first DMT* and *DMT switch* cohort. The observed 11.5 FSMC total score increase from moderate to severe fatigue trajectory at the end of follow-up in the *first DMT* cohort was clinically relevant (defined as ≥10 FSMC total scores).^36^ In contrast, the difference in FSMC total between DMT start and follow-up end for fatigue trajectories in the *DMT switch* cohort was unlikely to constitute a clinically meaningful change.

### Associations between fatigue and physical disability trajectories

**Figure 3:**
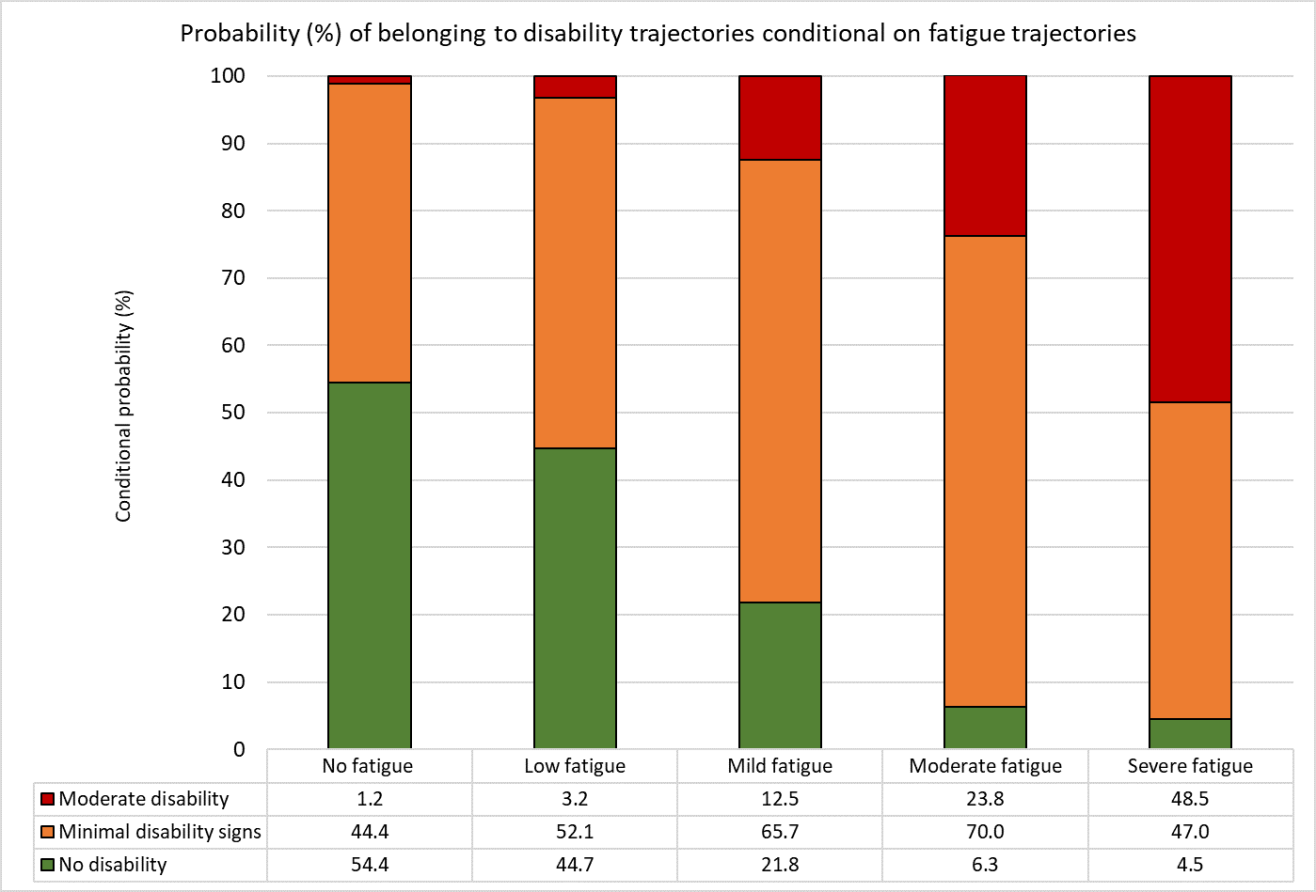
Probabilities of belonging to physical disability trajectories (Expanded Disability Status Scale) conditioned on belonging to each fatigue trajectory (Fatigue Scale for Motor and Cognitive Function), quantifying the association between fatigue and physical disability in relapsing–remitting multiple sclerosis people over 11 years following first disease-modifying therapy initiation.

We identified three EDSS-based physical disability trajectories, in each of the *first DMT* and *DMT switch* cohorts (online supplemental eFigure 1), strongly associated with fatigue trajectories. In the *first DMT* cohort (figure 3), the highest probability (48.5%) of belonging to the moderate physical disability trajectory was found among participants in the severe fatigue trajectory. The probability of belonging to the moderate physical disability trajectory stepwise decreased for participants in the moderate (23.8%), mild (12.5%), low (3.2%) and no (1.2%) fatigue trajectories, respectively. Similar associations between fatigue and physical disability trajectories were evident in the *DMT switch* cohort (online supplemental eFigure 2).

### Potential predictors of fatigue trajectories

Sociodemographic characteristics, comorbidities, treatment use and MS disease-related characteristics of the study participants in the *first DMT* cohort, stratified by the identified fatigue trajectories, are shown in table 1. Multinomial logistic regression analyses adjusting for all potential predictors, except baseline FSMC total, are shown in table 2. To visualise baseline fatigue’s impact on associations between potential predictors and fatigue trajectories, comparisons additionally adjusted for baseline FSMC total are in online supplemental eTable 3. Corresponding results for the *DMT switch* cohort are in online supplemental eTables 4–6.

**Table 1:** Characteristics of study participants at *first DMT* start according to fatigue trajectories (n=1587)

	FSMC total trajectories according to FSMC total starting values, n (%)				
	No	Low	Mild	Moderate	Severe
N (%)	288 (18.1)	382 (24.1)	340 (21.4)	320 (20.2)	257 (16.2)
Age at DMT start, years					
18–29	99 (34.4)	140 (36.6)	100 (29.4)	80 (25.0)	65 (25.3)
30–44	144 (50.0)	167 (43.7)	160 (47.1)	161 (50.3)	114 (44.4)
>44	45 (15.6)	75 (19.6)	80 (23.5)	79 (24.7)	78 (30.4)
Female	162 (56.3)	254 (66.5)	245 (72.1)	240 (75.0)	216 (84.0)
Born in Sweden	266 (92.4)	344 (90.1)	302 (88.8)	273 (85.3)	216 (84.0)
Education over 12 years	179 (62.4)	231 (60.8)	174 (51.2)	147 (46.1)	111 (43.2)
Sick leave previous year, mean (SD), days*	2.5 (30.1)	2.4 (27.2)	6.0 (39.5)	14.9 (67.0)	27.8 (88.4)
Disability pension previous year, mean (SD), days*	6.8 (17.9)	10.1 (34.1)	18.8 (45.0)	28.7 (62.7)	49.7 (81.3)
Comorbidity ≥1†	15 (5.2)	27 (7.1)	41 (12.1)	42 (13.1)	36 (14.0)
Depression diagnosis‡	1 (0.3)	7 (1.8)	12 (3.5)	31 (9.7)	35 (13.6)
Anxiety diagnosis‡	5 (1.7)	23 (6.0)	23 (6.8)	37 (11.6)	50 (19.5)
Other psychiatric comorbidities‡§	6 (2.1)	19 (5.0)	24 (7.1)	22 (6.9)	41 (16.0)
Antidepressants treatment¶	8 (2.8)	30 (7.9)	37 (10.9)	58 (18.1)	66 (25.7)
Anxiolytics treatment¶	6 (2.1)	15 (3.9)	7 (2.1)	15 (4.7)	20 (7.8)
Symptomatic fatigue treatment¶	1 (0.3)	2 (0.5)	9 (2.6)	6 (1.9)	9 (3.5)
Sleeping aids treatment¶	20 (6.9)	36 (9.4)	42 (12.4)	38 (11.9)	60 (23.3)
Pain treatment¶	71 (24.7)	96 (25.1)	97 (28.5)	115 (35.9)	128 (49.8)
DMT					
Dimethyl fumarate	61 (21.2)	75 (19.6)	65 (19.1)	49 (15.3)	46 (17.9)
Fingolimod	13 (4.5)	20 (5.2)	16 (4.7)	7 (2.2)	7 (2.7)
Glatiramer acetate	8 (2.8)	13 (3.4)	10 (2.9)	18 (5.6)	10 (3.9)
Interferons**	95 (33.0)	106 (27.7)	102 (30.0)	93 (29.1)	70 (27.2)
Natalizumab	30 (10.4)	66 (17.3)	50 (14.7)	49 (15.3)	42 (16.3)
Rituximab	77 (26.7)	88 (23.0)	89 (26.2)	93 (29.1)	73 (28.4)
Teriflunomide	4 (1.4)	14 (3.7)	8 (2.4)	11 (3.4)	9 (3.5)
MS duration (years)					
0–10	278 (96.9)	373 (97.9)	321 (94.7)	308 (96.6)	249 (97.3)
>10	9 (3.1)	8 (2.1)	18 (5.3)	11 (3.4)	7 (2.7)
Relapse previous year	215 (74.7)	252 (66.0)	221 (65.0)	204 (63.8)	177 (68.9)
New cerebral lesion previous year	109 (44.3)	171 (50.6)	138 (45.2)	141 (49.3)	113 (49.3)
FSMC total score, mean (SD)	23.1 (5.5)	34.7 (10.8)	46.9 (11.5)	56.4 (14.1)	78.1 (13.6)
EDSS score, median (IQR)	1.0 (0.0–2.0)	1.5 (1.0–2.0)	2.0 (1.0–2.5)	2.0 (1.0–2.5)	2.5 (2.0–3.0)
SDMT score, mean (SD)	56.5 (12.1)	55.7 (12.4)	52.4 (10.8)	51.1 (11.2)	47.7 (11.5)
MSIS-29 physical score, mean (SD)	5.8 (9.9)	9.5 (12.5)	18.4 (16.9)	23.4 (18.5)	41.8 (21.3)
MSIS-29 psychological score, mean (SD)	18.1 (18.5)	25.2 (19.5)	35.4 (22.3)	42.4 (21.4)	58.8 (21.6)
EQ-5D VAS score, mean (SD)	82.5 (16.1)	78.8 (17.2)	69.4 (18.2)	64.1 (18.2)	51.1 (22.4)
MS-symptoms inventory score, mean (SD)	2.2 (2.0)	5.6 (4.5)	6.8 (4.4)	9.8 (4.9)	14.3 (4.4)

* Restricted to participants 18–64 years old.

† Diagnosed within 5 years prior to DMT start according to the Charlson Comorbidity Index.

‡ Diagnosed within 5 years prior to DMT start.

§ All mental and behavioural disorders except depression and anxiety disorders.

¶ Dispensed prescribed drugs within 1 year prior to DMT start.

** Interferon beta-1a, peginterferon beta-1a and interferon beta-1b.

DMTdisease-modifying therapyEDSSExpanded Disability Status ScaleEQ-5D VASEuroQol Visual Analogue ScaleFSMCFatigue Scale for Motor and Cognitive functionMSmultiple sclerosisMSIS-29MS Impact ScaleSDMTSymbol Digit Modalities Test

**Table 2:** ORs of belonging to fatigue trajectories (compared with the no fatigue one) in a multivariable model including region of residence and excluding FSMC total, in addition to all potential predictors listed, n=1587 participants with RRMS on *first DMT*

	FSMC total trajectories according to FSMC total starting values			
	Low	Mild	Moderate	Severe
	OR (95% CI)	OR (95% CI)	OR (95% CI)	OR (95% CI)
Age, years				
18–29	Ref.	Ref.	Ref.	Ref.
30–44	0.94 (0.72 to 1.23)	1.10 (0.83 to 1.46)	1.33 (0.96 to 1.84)	1.26 (0.86 to 1.83)
>44	0.87 (0.58 to 1.32)	0.87 (0.57 to 1.35)	0.74 (0.46 to 1.19)	0.86 (0.49 to 1.51)
Female vs male	1.62 (1.08 to 2.43)	2.40 (1.56 to 3.69)	2.96 (1.78 to 4.92)	7.01 (3.65 to 13.48)
Born in Sweden vs born outside Sweden	0.87 (0.42 to 1.81)	0.70 (0.32 to 1.50)	0.64 (0.26 to 1.55)	0.53 (0.19 to 1.50)
Years of education >12 versus ≤12	1.18 (0.74 to 1.90)	0.90 (0.55 to 1.46)	0.78 (0.43 to 1.41)	0.91 (0.44 to 1.85)
Disability pension previous year*, days	1.00 (0.99 to 1.00)	1.00 (0.99 to 1.00)	1.00 (0.99 to 1.01)	1.00 (0.99 to 1.01)
Sick leave previous year*, days	1.00 (0.99 to 1.01)	1.00 (1.00 to 1.01)	1.01 (1.00 to 1.02)	1.01 (1.00 to 1.02)
Comorbidity ≥1† vs none	1.10 (0.45 to 2.71)	1.80 (0.75 to 4.33)	1.50 (0.51 to 4.45)	0.90 (0.27 to 3.07)
History of depression‡, yes vs no	2.47 (0.21 to 28.73)	4.04 (0.35 to 46.17)	14.89 (1.17 to 189.50)	11.51 (0.85 to 155.96)
History of anxiety‡, yes vs no	2.94 (0.80 to 10.79)	1.82 (0.47 to 7.04)	2.19 (0.50 to 9.54)	3.03 (0.60 to 15.15)
History of other psychiatric comorbidities‡§, yes vs no	1.27 (0.36 to 4.50)	1.57 (0.45 to 5.50)	0.75 (0.18 to 3.11)	1.91 (0.46 to 7.99)
History of antidepressants treatment¶, yes vs no	1.90 (0.66 to 5.46)	2.40 (0.81 to 7.13)	2.97 (0.91 to 9.69)	3.04 (0.87 to 10.58)
History of anxiolytics treatment¶, yes vs no	1.00 (0.27 to 3.78)	0.27 (0.06 to 1.19)	0.47 (0.09 to 2.37)	0.43 (0.07 to 2.56)
History of symptomatic fatigue treatment¶, yes vs no	0.52 (0.02 to 11.97)	1.28 (0.07 to 23.24)	0.58 (0.02 to 16.86)	0.81 (0.02 to 27.32)
History of sleeping aids treatment¶, yes vs no	0.54 (0.23 to 1.28)	0.54 (0.22 to 1.35)	0.25 (0.09 to 0.68)	0.31 (0.10 to 0.97)
History of pain treatment¶, yes vs no	1.06 (0.63 to 1.77)	1.12 (0.66 to 1.92)	1.52 (0.80 to 2.88)	2.39 (1.16 to 4.96)
DMT				
Rituximab	Ref.	Ref.	Ref.	Ref.
Dimethyl fumarate	0.82 (0.48 to 1.40)	0.96 (0.55 to 1.69)	0.79 (0.38 to 1.62)	0.96 (0.42 to 2.19)
Fingolimod	1.10 (0.49 to 2.48)	1.09 (0.46 to 2.61)	0.45 (0.14 to 1.46)	0.65 (0.17 to 2.52)
Glatiramer acetate	1.27 (0.39 to 4.13)	1.07 (0.29 to 3.95)	2.50 (0.51 to 12.17)	1.93 (0.31 to 12.03)
Interferon**	0.29 (0.13 to 0.66)	0.40 (0.19 to 0.87)	0.24 (0.09 to 0.66)	0.21 (0.08 to 0.61)
Natalizumab	1.52 (0.85 to 2.73)	1.26 (0.67 to 2.38)	1.18 (0.56 to 2.49)	0.90 (0.38 to 2.17)
Teriflunomide	3.43 (1.02 to 11.54)	2.82 (0.77 to 10.32)	5.71 (1.28 to 25.42)	8.13 (1.53 to 43.08)
MS duration >10 years versus ≤10 years	0.69 (0.35 to 1.34)	1.09 (0.56 to 2.12)	0.79 (0.35 to 1.80)	0.74 (0.27 to 2.04)
Any relapse vs none in the previous year	0.70 (0.44 to 1.11)	0.63 (0.39 to 1.02)	0.70 (0.39 to 1.24)	0.83 (0.43 to 1.60)
Any new cerebral lesions vs none in the previous year	1.12 (0.72 to 1.74)	0.87 (0.54 to 1.40)	0.97 (0.55 to 1.71)	0.76 (0.37 to 1.53)
FSMC total score	1.14 (1.05 to 1.24)	1.24 (1.14 to 1.34)	1.32 (1.22 to 1.43)	1.47 (1.32 to 1.65)
EDSS score	1.02 (0.81 to 1.30)	1.21 (0.92 to 1.59)	1.08 (0.78 to 1.48)	1.14 (0.77 to 1.67)
SDMT score	0.99 (0.97 to 1.02)	0.98 (0.96 to 1.01)	0.98 (0.95 to 1.01)	0.97 (0.94 to 1.01)
MSIS-29 physical score	0.96 (0.93 to 1.00)	0.99 (0.95 to 1.03)	0.97 (0.93 to 1.01)	1.01 (0.97 to 1.06)
MSIS-29 psychological score	1.01 (0.99 to 1.03)	1.02 (1.00 to 1.04)	1.02 (1.00 to 1.05)	1.03 (1.01 to 1.06)
EQ-5D VAS score	1.02 (1.00 to 1.04)	1.00 (0.98 to 1.02)	1.00 (0.98 to 1.03)	1.01 (0.98 to 1.03)
MS-symptoms inventory score	1.49 (1.20 to 1.87)	1.45 (1.13 to 1.86)	1.76 (1.41 to 2.20)	1.79 (1.36 to 2.36)

* Restricted to participants 18–64 years old.

† Diagnosed within 5 years prior to DMT start according to the Charlson Comorbidity Index.

‡ Diagnosed within 5 years prior to DMT start.

§ All mental and behavioural disorders except depression and anxiety disorders.

¶ Dispensed prescribed drugs within 1 year prior to DMT start.

** Interferon beta-1a, peginterferon beta-1a and interferon beta-1b.

DMTdisease-modifying therapyEDSSExpanded Disability Status ScaleEQ-5D VASEuroQol Visual Analogue ScaleFSMCFatigue Scale for Motor and Cognitive FunctionMSmultiple sclerosisMSIS-29MS Impact ScaleSDMTSymbol Digit Modalities Test

After mutual adjustment for potential predictors, excluding FSMC total, we found that female sex was associated with higher odds of belonging to low to severe fatigue trajectories, relative to the no fatigue group (table 2). Among MS-related characteristics, higher scores on the MSIS-29 psychological and MS-symptoms inventory were associated with higher odds of reporting greater fatigue. Participants starting teriflunomide as first DMT were more likely to belong to the severe, moderate and low fatigue trajectory, relative to the no fatigue one, than participants starting rituximab. Notably, a history of prescribed pain medications was linked to higher odds of fatigue, while pharmacological sleeping aids were unexpectedly associated with lower odds of belonging to a more pronounced fatigue trajectory group (table 2). History of depression, but not anxiety, increased the risk of a more pronounced fatigue trajectory, while antidepressants use showed a non-significant trend for increased risk (table 2). Additional adjustment for baseline FSMC total scores revealed that the association between higher MSIS-29 psychological scores and more pronounced fatigue was lost, while an inverse association to higher MSIS-29 physical scores emerged (online supplemental eTable 3). Comparable associations were found in the *DMT switch* cohort, with a few differences (online supplemental eTables 4–6). Associations with history of pain and sleeping aids treatments were no longer evident. However, increased sick leave days in the last year were associated with higher probability of belonging to the low, mild, moderate and severe fatigue trajectory groups, relative to the no fatigue group.

### DMT switch analysis

Out of 1587 participants in the *first DMT* cohort, 867 belonged to the *first line DMT escalation* subcohort. Among these, 619 (71%) participants switched to an escalation DMT during follow-up, while 248 (29%) switched to another first-line DMT or stayed on the same DMT within the subcohort. Incorporating a switch to an escalation DMT as a time-varying covariate at all relevant points showed a statistically significant, although minor, increase in low, mild and severe fatigue trajectories. This suggests a potential association between switching to an escalation DMT and increased fatigue in these trajectories (online supplemental eFigures 3–11). The Wald test did not reveal any statistically significant differential effect of DMT switch by fatigue trajectory group when using no fatigue trajectory group as reference.

### Sensitivity analyses

Findings were similar across FSMC cognitive and motor subdomains (online supplemental eTables 7–10, online supplemental eFigures 12 and 13). Excluding participants with fatigue trajectory assignment probability <0.80 (n=268 in the *first DMT* cohort, n=348 in the *DMT switch* cohort) rendered similar predictors of fatigue trajectories (online supplemental eTables 11 and 12).

## Discussion

In this large population-based cohort of actively treated RRMS people, we observe mostly stable self-reported fatigue trajectories over 11 years from DMT start. The only exception was the moderate fatigue trajectory at *first DMT* start, which increased from moderate to severe fatigue. We also found a strong association between higher fatigue scores and physical disability.

The finding of stable or moderately increasing fatigue scores is partly consistent with the majority of previous studies, which have reported stable scores over time.^9^,^13^ In contrast to this study, the follow-up time in these studies was generally short, ranging from 1 to 3 years, which does not allow for fluctuations over a longer period of time. Another study, which included 944 individuals with RRMS, with a median of 2 years from diagnosis to baseline and a median follow-up of 8 years, reported sustained fatigue worsening among 52% of the study population, defining fatigue worsening as a ≥1-point increase in the Fatigue Performance Scale (FPS).^8^ While this study observed that worsening of fatigue was associated with lower levels of fatigue at baseline, we observed persistent fatigue worsening only among people with moderate fatigue at first DMT start, with stable fatigue trajectories in other fatigue groups. Although similarly to our study, this study followed individuals from early in the disease course, the difference could possibly be attributed to how fatigue was conceptualised. While FSMC aims to capture several dimensions of fatigue, the FPS is a single-item scale measuring global fatigue.^8^ Among studies following individuals from initiation of a specific DMT, the majority suggest reduced fatigue scores over time.^5^,^7^ However, these studies were characterised by short follow-up periods. Studies employing latent class modelling to address depression score trajectories in MS have also shown relatively stable patterns over time,^37^ despite depression being typically viewed as an episodic disorder. This underscores the difficulty in capturing smaller variations in longitudinal studies, which require short intervals between follow-up measures.

Unlike prior longitudinal studies,^6 10^ we also find a strong association between fatigue and worsening of physical disability, although temporal relation and direction of potential causality remain uncertain. Further, existing literature indicates that fatigue is commonly experienced also in early MS with low disability.^13 38^ This observation is corroborated here, but expands on identifying baseline fatigue as a strong predictor of fatigue trajectory membership. Collectively, this indicates that the relation between physical disability and self-reported fatigue is complex and that the origin of fatigue can vary between and possibly within PwMS over the course of the disease.^3^ As such inflammatory disease activity has been suggested an important factor in earlier stages, while accumulated injury, neurodegeneration and comorbidities dominate later on.^39^ It should be noted, however, that start of DMT should have reduced fatigue severity if inflammation constituted a relevant driver of fatigue, therefore instead pointing at other factors being important in the early disease course.

Unexpectedly, however, only a few factors independently predicted membership in the higher-stable or moderately increasing fatigue trajectories. Aligning with prior cross-sectional data in partly overlapping cohorts,^2^ we found that female sex, productivity loss, MSIS-29 psychological domain and MS-symptoms inventory scores (both of which includes questions about fatigue) were linked to reporting of fatigue. Adjustment for baseline fatigue strongly diminished associations with fatigue trajectories, and the stepwise addition of covariates exerted a strong effect on final results. For instance, numerous studies have shown an association between depression and fatigue,^914^,^16^ and we here also observed notable differences in history of depression diagnosis and antidepressant use in unadjusted comparisons. However, these associations lost statistical significance on adjusting for other predictors, implying that the relation is confounded or influenced in some other manner by factors related to MS. Moreover, our results showed that individuals initiating teriflunomide are more likely to have higher fatigue levels already at DMT start compared with those starting rituximab, but may also indicate a potential inferiority regarding later fatigue evolution relative to rituximab. However, wide CIs, especially for teriflunomide, means that results should be interpreted with caution. Interestingly, however, a recent randomized controlled trial showed superiority of ponesimod over teriflunomide regarding fatigue,^40^ although differences were relatively small. Taken together, existing data therefore suggest that impact of DMT is likely to be relatively modest, at least in the medium term.

In line with other studies examining fatigue changes over time from diagnosis of MS,^8 13^ we observed that a majority reported fatigue already at first DMT start. Further, degree of fatigue at DMT start emerged as a strong predictor for evolution of fatigue scores later on, indicating that this symptom tends to be sustained over time. As discussed previously, also factors not relating to MS-specific disease characteristics could play an important role, some of which we could not capture here, such as physical deconditioning^3^ or psychological responses to being diagnosed with a chronic disease. Nevertheless, at the individual level, it is clear that fatigue impacts quality of life and capacity to engage in desired daily activities. Therefore, in a clinical setting, it is important to thoroughly investigate, rule out and treat potential non-disease-specific factors contributing to fatigue.

### Strengths and limitations

Our study’s major strengths include population-based setting, a large sample size, extended duration and a comprehensive set of high-quality prospectively collected covariates. We also studied and compared two common and distinct RRMS scenarios, that is, first start and switch of DMT. The application of group-based trajectory modelling, considering between-person variation,^33^ and heterogeneity of fatigue and physical disability over time, is an additional strength. We avoided adding random effects into the model to capture maximum population variability, potentially leading to a higher number of identified trajectory groups.

The study has limitations. First, some covariates, especially imaging and MS-specific scales, had high data missingness, potentially introducing bias with multiple imputations. However, main results aligned with complete-case analyses, indicating limited bias. Second, while we considered the effect of DMT switch, we could not discern if increased fatigue resulted directly from the switch influenced the decision to switch. We also did not account for DMT discontinuation, nor did we interpret our results in light of a possible long-term effect of specific DMTs on fatigue trajectories. Third, there is no consensus on the optimal patient-reported outcome instrument for assessing fatigue in MS, and different instruments conceptualise fatigue differently. In our study, we used the FSMC to measure fatigue, given that it was developed specifically for MS and its well-established usage in the Swedish MS community, but also with increasing use at the international level. The FSMC is relatively new and less commonly employed compared with more established scales such as the Modified Fatigue Impact Scale (MFIS) or the Fatigue Severity Scale (FSS). Although FSMC demonstrates a strong correlation with the MFIS (r=0.83) and the FSS (r=0.80),^24^ the comparability of our findings with other studies that used these two scales are limited. Fourth, given the relatively high number of statistical analyses conducted in the analyses of potential predictors of fatigue trajectories, caution is warranted when interpreting these results. Lastly, we lacked information on potentially relevant life style factors such as physical activity^3^ and smoking, as well as sleep disorders.^4^

## Conclusions

Over an average follow-up period of 7 years following the initiation of either first ever DMT or first ever DMT switch, we observed that fatigue ratings in RRMS people who already had a substantial burden of fatigue symptoms at the start of DMT either remained stable or increased. Our findings emphasise the critical role of early fatigue screening in managing fatigue symptoms or ruling out non-disease-specific factors as contributor to fatigue. Further research is needed to investigate the impact of different DMTs and symptomatic fatigue treatments on fatigue in individuals with MS.

## supplementary material

10.1136/jnnp-2024-333595 online supplemental file 1

## Data Availability

Data may be obtained from a third party and are not publicly available.
