# Supplementary material for: Trajectories of self-reported fatigue following initiation of multiple sclerosis disease-modifying therapy
Source: J Neurol Neurosurg Psychiatry. 2024 May 14;95(11):e333595. doi: 10.1136/jnnp-2024-333595 (PMC11503085; doi:10.1136/jnnp-2024-333595)
Supplement: online supplemental file 1 [file jnnp-95-11-s001.docx]

Supplementary material for

Trajectories of self-reported fatigue following initiation of multiple sclerosis disease-modifying therapy

Contents

[eTable 1 2](#_Toc161219371)

[eTable 2 5](#_Toc161219372)

[eTable 3 6](#_Toc161219373)

[eTable 4 8](#_Toc161219374)

[eTable 5 10](#_Toc161219375)

[eTable 6 12](#_Toc161219376)

[eTable 7 14](#_Toc161219377)

[eTable 8 16](#_Toc161219378)

[eTable 9 18](#_Toc161219379)

[eTable 10 20](#_Toc161219380)

[eTable 11 22](#_Toc161219381)

[eTable 12 24](#_Toc161219382)

[Figures 26](#_Toc161219383)

[eFigure 1 26](#_Toc161219384)

[eFigure 2 27](#_Toc161219385)

[eFigure 3. 28](#_Toc161219386)

[eFigure 4. 28](#_Toc161219387)

[eFigure 5 29](#_Toc161219388)

[eFigure 6. 29](#_Toc161219389)

[eFigure 7 30](#_Toc161219390)

[eFigure 8. 30](#_Toc161219391)

[eFigure 9. 31](#_Toc161219392)

[eFigure 10. 31](#_Toc161219393)

[eFigure 11. 32](#_Toc161219394)

[eFigure 12. 33](#_Toc161219395)

[eFigure 13. 34](#_Toc161219396)

[References for supplementary material 35](#_Toc161219397)

Tables

eTable 1: Description and data source of potential predictors of fatigue trajectories included in the analyses

| **Variable** | **Description** | **Data source** |
| --- | --- | --- |
| **Socio-demographic** |  |  |
| Age at DMT start | Age at DMT start. Categorized into: 18-29, 30-44, >44 years. | Total Population Register |
| Sex | Categorized into: female, male. | Total Population Register |
| Region of residence | Geographical region of residence at DMT start. Categorized into: Northern, Middle, Western South-eastern, Southern Sweden, Stockholm. | MS Registry |
| Country of birth | Categorized into: Sweden, outside Sweden. | Total Population Register |
| Education | Highest education achieved as recorded in the year prior to DMT start. Categorized into: ≤12 years or >12 years of education. | LISA |
| **Productivity loss** |  |  |
| History of sick leave | Number of sick leave days within one year prior to inclusion DMT start. Continuous variable. Restricted to participants 18-64 years old. | MiDAS |
| History of disability pension | Number of disability pension days within one year prior to inclusion DMT start. Continuous variable. Restricted to participants 18-64 years old. | MiDAS |
| **Comorbidity** |  |  |
| CCI | CCI adapted for register-based research in Sweden^1^. It includes myocardial infarction, congestive heart failure, peripheral vascular disease, cerebrovascular disease, pulmonary diseases, rheumatic disease, dementia, hemiplegia, diabetes, chronic kidney disease, liver disease, peptic ulcer disease, cancer and HIV/AIDS. Calculated using comorbidities diagnosed in the 5 years prior to DMT start. Categorized into: 0 or ≥1 CCI score. | Prescribed Drug Register and National Patient Register |
| History of depression | History of depression recorded in the 5 years prior to DMT start. Defined as a record in the National Patient Register (inpatient and outpatient components, ICD10: F32-F34, F38-F39). Indicator variable (Y/N). | National Patient Register |
| History of anxiety disorders | History of anxiety disorders recorded in the 5 years prior to DMT start. Defined as a record in the National Patient Register (inpatient and outpatient components, ICD10: F40-F45, F48). Indicator variable (Y/N). | National Patient Register |
| History of other psychiatric comorbidities | History of other mental or behavioral disorder recorded in the 5 years prior to DMT start. Defined as a record in the National Patient Register (inpatient and outpatient components, F00-F99 except F32-34, F38-39 and F40-48). Indicator variable (Y/N). | National Patient Register |
| **Treatment dispensation** |  |  |
| History of antidepressants treatment | History of dispensed antidepressants recorded in the year prior to DMT start. Defined as a record in the Prescribed Drug Register (ATC: N06AB, N06AF, N06AG, N06AX but not N06AX21. N06AA and N06AX21 not included as commonly used to treat neuropathic pain in people with MS). Indicator variable (Y/N). | Prescribed Drug Register |
| History of anxiolytics treatment | History of dispensed anxiolytics recorded in the year prior to DMT start. Defined as a record in the Prescribed Drug Register (ATC: N05BA12, N05BA06, 05BA06. N05BA01 not included as commonly used to treat other symptoms in people with MS). Indicator variable (Y/N). | Prescribed Drug Register |
| History of symptomatic treatment for fatigue | History of dispensed central stimulants recorded in the year prior to DMT start. Defined as a record in the Prescribed Drug Register (ATC: N06BA01, N06BA02, N06BA04, N06BA07, N06BA09, N06BA12). Indicator variable (Y/N). | Prescribed Drug Register |
| History of sleeping aids treatment | History of dispensed sleeping aids recorded in the year prior to DMT start. Defined as a record in the Prescribed Drug Register (ATC: N05CF01, N05CF02, N05CF03, N05CM02, N05CM06, N05CM09, N05CD02, N05CD05). Indicator variable (Y/N). | Prescribed Drug Register |
| History of pain treatment | History of dispensed pain treatment recorded in the year prior to DMT start. Defined as a record in the Prescribed Drug Register (ATC: N02A, N02BE, M03BB, M03BC, N02BA, N03AX12, N03AX16, N02C, N02BG10, N06AA09, N06AA10). Indicator variable (Y/N). | Prescribed Drug Register |
| **MS disease-related** |  |  |
| DMT | First DMT cohort: dimethyl fumarate, fingolimod, glatiramer acetate, interferons (interferon beta-1a, peginterferon beta-1a, and interferon beta-1b), natalizumab, rituximab, or teriflunomide.  DMT switch cohort: dimethyl fumarate, fingolimod, natalizumab, rituximab, or teriflunomide. | MS Registry |
| MS duration | Number of years from MS diagnosis at DMT start. Categorized into: 0-10, >10. | MS Registry |
| Relapse | Any relapse within one year prior to DMT start. Indicator variable (Y/N). | MS Registry |
| New cerebral lesions | Any new cerebral gadolinium-enhancing lesions in the year prior to DMT start. Indicator variable (Y/N). | MS Registry |
| Fatigue | Fatigue assessed via FSMC total score recorded at the most recent visit prior to DMT start (within -30 to 15 days from DMT start). Continuous variable. | MS Registry |
| Physical disability | Physical disability assessed via EDSS recorded at the most recent visit prior to DMT start (within -180 to 15 days from DMT start). Continuous variable. | MS Registry |
| Processing speed | Processing assessed via SDMT recorded at the most recent visit prior to DMT start (within -30 to 15 days from DMT start). Continuous variable. | MS Registry |
| Physical impact of MS | Physical impact of MS assessed via MSIS-29 physical recorded at the most recent visit prior to DMT start (within -30 to 15 days from DMT start). Continuous variable. | MS Registry |
| Psychological impact of MS | Psychological impact of MS assessed via MSIS-29 physical recorded at the most recent visit prior to DMT start (within -30 to 15 days from DMT start). Continuous variable. | MS Registry |
| Health-related quality of life | Self-rated health assessed via EQ-5D VAS recorded at the most recent visit prior to DMT start (within -30 to 15 days from DMT start). Continuous variable. | MS Registry |
| Self-reported disability | Disability assessed via the MS-symptoms inventory scale (a modification of the the Guy's Neurological Disability Scale; GNDS^2^) recorded at the most recent visit prior to DMT start (within -30 to 15 days from DMT start). Continuous variable. | MS Registry |
| AIDS, acquired immunodeficiency syndrome; ATC, anatomical therapeutic chemical; CCI, Charlson comorbidity index; DMT, disease-modifying therapy; EDSS, Expanded Disability Status Scale; EQ-5D VAS, EuroQol Visual Analogue Scale; FSMC, Fatigue Scale for Motor and Cognitive function; HIV, human immunodeficiency virus; ICD, international classification of disease; LISA, the longitudinal integrated database for health Insurance and labour market studies; MiDAS, the Swedish social insurance agency; MS, multiple sclerosis; MSIS- 29, MS Impact Scale; SDMT, Symbol Digit Modalities Test. | | |

| eTable 2: Frequency of missing values at first DMT and DMT switch start of potential predictors included in the analyses, N=1,587 participants at first DMT start and 1,818 participants at DMT switch start | | |
| --- | --- | --- |
|  | **Missing values at DMT start** | |
|  | **First line DMT** | **First second line DMT** |
| **Covariates** | **N (%)** | **N (%)** |
| Age at DMT start (years) | 0 (0.0) | 0 (0.0) |
| Female | 0 (0.0) | 0 (0.0) |
| Region of residence | 0 (0.0) | 0 (0.0) |
| Born in Sweden | 0 (0.0) | 0 (0.0) |
| Education (years) | 4 (0.3) | 2 (0.1) |
| Sick leave previous year^a^ | 0 (0.0) | 0 (0.0) |
| Disability pension previous year^a^ | 0 (0.0) | 0 (0.0) |
| Comorbidity ≥ 1^b^ | 0 (0.0) | 0 (0.0) |
| Depression diagnosis^c^ | 0 (0.0) | 0 (0.0) |
| Anxiety diagnosis^c^ | 0 (0.0) | 0 (0.0) |
| Other psychiatric comorbidities^c,d^ | 0 (0.0) | 0 (0.0) |
| Antidepressants treatment^e^ | 0 (0.0) | 0 (0.0) |
| Anxiolytics treatment^e^ | 0 (0.0) | 0 (0.0) |
| Symptomatic fatigue treatment^e^ | 0 (0.0) | 0 (0.0) |
| Sleeping aids treatment^e^ | 0 (0.0) | 0 (0.0) |
| Pain treatment^e^ | 0 (0.0) | 0 (0.0) |
| DMT | 0 (0.0) | 0 (0.0) |
| MS duration (years) | 5 (0.3) | 4 (0.2) |
| Relapses | 0 (0.0) | 0 (0.0) |
| New cerebral lesion | 183 (11.5) | 291 (15.9) |
| FSMC total Score | 1313 (82.7) | 1595 (86.9) |
| EDSS Score | 306 (19.3) | 401 (21.9) |
| SDMT Score | 747 (47.1) | 467 (25.4) |
| MSIS-29 Physical Score | 704 (44.4) | 444 (24.2) |
| MSIS-29 Psychological Score | 704 (44.4) | 444 (24.2) |
| EQ-5D VAS Score | 816 (51.4) | 708 (38.6) |
| MS-symptoms inventory Score | 1313 (82.7) | 1590 (86.6) |
| ^a^Restricted to participants 18-64 years old.  ^b^Diagnosed within 5 years prior to DMT start according to the Charlson Comorbidity Index.  ^c^Diagnosed within five years prior to DMT start.  ^d^All mental and behavioral disorders except depression and anxiety disorders.  ^e^Dispensed prescribed drugs within 1 year prior to DMT start.  DMT, disease-modifying therapy; EDSS, Expanded Disability Status Scale; EQ-5D VAS, EuroQol Visual Analogue Scale; FSMC, Fatigue Scale for Motor and Cognitive function; MS, multiple sclerosis; MSIS- 29, MS Impact Scale; SDMT, Symbol Digit Modalities Test. | | |

| eTable 3 ORs of belonging to FSMC total trajectories (compared with the FSMC total one) in a multivariable model including region of residence and FSMC total, in addition to all potential predictors listed, N=1,587 participants with RRMS on *first DMT* | | | | | |
| --- | --- | --- | --- | --- | --- |
|  | **FSMC total trajectories according to FSMC total starting values** | | | | |
|  | | **Low** | **Mild** | **Moderate** | **Severe** |
|  | | **OR (95% CI)** | **OR (95% CI)** | **OR (95% CI)** | **OR (95% CI)** |
| Age, years | |  |  |  |  |
| 18-29 | | Ref. | Ref. | Ref. | Ref. |
| 30-44 | | 0.88 (0.63; 1.22) | 0.96 (0.65; 1.43) | 1.11 (0.69; 1.79) | 0.99 (0.51; 1.91) |
| >44 | | 0.87 (0.55; 1.37) | 0.85 (0.46; 1.57) | 0.69 (0.34; 1.39) | 0.70 (0.26; 1.86) |
| Female versus Male | | 1.48 (0.90; 2.45) | 1.94 (0.98; 3.84) | 2.24 (0.99; 5.06) | 4.85 (1.50; 15.72) |
| Born in Sweden versus born outside Sweden | | 1.03 (0.44; 2.42) | 0.92 (0.32; 2.59) | 0.92 (0.27; 3.17) | 0.94 (0.20; 4.49) |
| Years of education>12 versus ≤12 | | 1.24 (0.76; 2.03) | 0.99 (0.53; 1.84) | 0.87 (0.41; 1.84) | 0.97 (0.37; 2.51) |
| Disability pension previous year^a^, days | | 1.00 (0.99; 1.01) | 1.00 (0.99; 1.01) | 1.00 (0.99; 1.02) | 1.00 (0.99; 1.02) |
| Sick leave previous year^a^, days | | 1.00 (0.99; 1.01) | 1.00 (0.99; 1.01) | 1.00 (0.99; 1.02) | 1.00 (0.99; 1.02) |
| Comorbidity≥1^b^ versus none | | 1.08 (0.41; 2.84) | 2.00 (0.65; 6.19) | 1.83 (0.47; 7.22) | 1.23 (0.21; 7.34) |
| History of depression^c^, yes versus no | | 2.08 (0.11; 40.74) | 2.59 (0.09; 71.40) | 8.13 (0.26; 259.01) | 3.74 (0.08; 165.06) |
| History of anxiety^c^, yes versus no | | 4.35 (0.93; 20.32) | 3.38 (0.57; 19.94) | 4.60 (0.64; 33.32) | 8.81 (0.94; 82.32) |
| History of other psychiatric comorbidities^c,d^, yes versus no | | 1.23 (0.27; 5.60) | 1.67 (0.28; 9.85) | 0.89 (0.11; 7.47) | 2.74 (0.23; 32.58) |
| History of antidepressants treatment^e^, yes versus no | | 1.73 (0.48; 6.19) | 2.03 (0.45; 9.09) | 2.22 (0.41; 12.18) | 1.68 (0.22; 12.86) |
| History of anxiolytics treatment^d^, yes versus no | | 1.58 (0.28; 9.01) | 0.61 (0.06; 6.23) | 1.57 (0.10; 25.61) | 3.13 (0.07; 137.82) |
| History of symptomatic fatigue treatment^e^, yes versus no | | 0.18 (0.00; 8.50) | 0.27 (0.00; 21.48) | 0.08 (0.00; 18.03) | 0.06 (0.00; 30.56) |
| History of sleeping aids treatment^e^, yes versus no | | 0.49 (0.16; 1.47) | 0.43 (0.10; 1.89) | 0.19 (0.03; 1.06) | 0.22 (0.02; 1.99) |
| History of pain treatment^e^, yes versus no | | 1.15 (0.64; 2.08) | 1.36 (0.66; 2.81) | 1.95 (0.81; 4.70) | 3.56 (1.05; 12.10) |
| DMT | |  |  |  |  |
| Rituximab | | Ref. | Ref. | Ref. | Ref. |
| Dimethyl fumarate | | 0.95 (0.46; 1.95) | 1.28 (0.52; 3.15) | 1.17 (0.36; 3.83) | 1.62 (0.34; 7.66) |
| Fingolimod | | 1.00 (0.32; 3.11) | 0.86 (0.14; 5.13) | 0.31 (0.03; 3.21) | 0.41 (0.01; 12.51) |
| Glatiramer acetate | | 1.05 (0.17; 6.58) | 0.79 (0.05; 12.18) | 1.70 (0.05; 58.62) | 1.31 (0.01; 162.19) |
| Interferon^f^ | | 0.30 (0.10; 0.95) | 0.43 (0.07; 2.69) | 0.27 (0.02; 3.38) | 0.24 (0.01; 8.08) |
| Natalizumab | | 1.86 (0.77; 4.50) | 1.74 (0.46; 6.62) | 1.76 (0.34; 9.06) | 1.48 (0.16; 13.65) |
| Teriflunomide | | 2.79 (0.52; 14.99) | 1.92 (0.19; 19.62) | 3.53 (0.21; 59.17) | 4.20 (0.09; 206.44) |
| MS duration>10 years versus ≤10 years | | 0.61 (0.28; 1.32) | 0.93 (0.38; 2.31) | 0.65 (0.21; 2.05) | 0.58 (0.12; 2.87) |
| Any relapse versus none in the previous year | | 0.68 (0.40; 1.16) | 0.58 (0.29; 1.16) | 0.64 (0.27; 1.51) | 0.74 (0.22; 2.50) |
| Any new cerebral lesions versus none in the previous year | | 1.01 (0.59; 1.74) | 0.71 (0.34; 1.49) | 0.73 (0.30; 1.78) | 0.42 (0.12; 1.48) |
| FSMC total Score | | 1.14 (1.05; 1.24) | 1.24 (1.14; 1.34) | 1.32 (1.22; 1.43) | 1.47 (1.32; 1.65) |
| EDSS Score | | 1.15 (0.85; 1.56) | 1.48 (1.01; 2.16) | 1.39 (0.89; 2.16) | 1.59 (0.84; 3.00) |
| SDMT Score | | 1.00 (0.97; 1.03) | 0.99 (0.96; 1.03) | 0.98 (0.94; 1.03) | 0.98 (0.94; 1.03) |
| MSIS-29 Physical Score | | 0.93 (0.89; 0.98) | 0.94 (0.89; 0.99) | 0.91 (0.86; 0.96) | 0.93 (0.86; 1.00) |
| MSIS-29 Psychological Score | | 1.00 (0.98; 1.02) | 0.99 (0.97; 1.02) | 0.99 (0.96; 1.02) | 0.98 (0.94; 1.02) |
| EQ-5D VAS Score | | 1.02 (1.00; 1.05) | 1.01 (0.98; 1.04) | 1.01 (0.98; 1.05) | 1.02 (0.98; 1.06) |
| MS-symptoms inventory Score | | 1.40 (1.12; 1.74) | 1.29 (1.00; 1.65) | 1.52 (1.21; 1.91) | 1.49 (1.08; 2.04) |
| ^a^Restricted to participants 18-64 years old.  ^b^Diagnosed within 5 years prior to DMT start according to the Charlson Comorbidity Index.  ^c^Diagnosed within five years prior to DMT start.  ^d^All mental and behavioral disorders except depression and anxiety disorders.  ^e^Dispensed prescribed drugs within 1 year prior to DMT start.  ^f^Interferon beta-1a, peginterferon beta 1 a, and interferon beta-1b.  DMT, disease-modifying therapy; EDSS, Expanded Disability Status Scale; EQ-5D VAS, EuroQol Visual Analogue Scale; FSMC, Fatigue Scale for Motor and Cognitive function; MS, Multiple Sclerosis; MSIS-29, MS Impact Scale; OR, Odds Ratio; SDMT, Symbol Digit Modalities Test. | | | | | |

| eTable 4 Characteristics of study participants at *DMT switch* start according to FSMC total trajectories (N=1,818) | | | | | | |
| --- | --- | --- | --- | --- | --- | --- |
|  | **FSMC total trajectories according to FSMC total starting values, N (%)** | | | | | |
|  | **No** | **Low** | **Mild** | **Moderate** | **Moderate/severe** | **Severe** |
| N (%) | 192 (10.6) | 364 (20.0) | 361 (19.9) | 347 (19.1) | 351 (19.3) | 203 (11.2) |
| Age at DMT start, years |  |  |  |  |  |  |
| 18-29 | 37 (19.3) | 80 (22.0) | 70 (19.4) | 62 (17.9) | 48 (13.7) | 37 (18.2) |
| 30-44 | 98 (51.0) | 204 (56.0) | 186 (51.5) | 158 (45.5) | 168 (47.9) | 96 (47.3) |
| >44 | 57 (29.7) | 80 (22.0) | 105 (29.1) | 127 (36.6) | 135 (38.5) | 70 (34.5) |
| Female | 121 (63.0) | 236 (64.8) | 248 (68.7) | 268 (77.2) | 287 (81.8) | 182 (89.7) |
| Born in Sweden | 182 (94.8) | 332 (91.2) | 325 (90.0) | 309 (89.0) | 296 (84.3) | 164 (80.8) |
| Education over 12 years | 121 (63.0) | 240 (65.9) | 198 (54.8) | 177 (51.0) | 164 (47.0) | 86 (42.4) |
| Sick leave previous year, mean (SD), days^a^ | 4.3 (31.6) | 7.4 (43.7) | 15.6 (58.5) | 34.7 (89.2) | 47.6 (103.3) | 71.2 (124.1) |
| Disability pension previous year, mean (SD), days^a^ | 2.3 (8.5) | 6.4 (20.8) | 16.6 (46.1) | 32.4 (70.4) | 43.7 (79.1) | 72.3 (104.6) |
| Comorbidity≥1^b^ | 11 (5.7) | 21 (5.8) | 29 (8.0) | 34 (9.8) | 46 (13.1) | 25 (12.3) |
| Depression diagnosis^c^ | 1 (0.5) | 7 (1.9) | 17 (4.7) | 19 (5.5) | 30 (8.5) | 21 (10.3) |
| Anxiety diagnosis^c^ | 3 (1.6) | 14 (3.8) | 19 (5.3) | 26 (7.5) | 36 (10.3) | 27 (13.3) |
| Other psychiatric comorbidities^c,d^ | 4 (2.1) | 5 (1.4) | 21 (5.8) | 25 (7.2) | 27 (7.7) | 20 (9.9) |
| Antidepressants treatment^e^ | 11 (5.7) | 31 (8.5) | 57 (15.8) | 74 (21.3) | 93 (26.5) | 72 (35.5) |
| Anxiolytics treatment^e^ | 2 (1.0) | 9 (2.5) | 12 (3.3) | 12 (3.5) | 23 (6.6) | 18 (8.9) |
| Symptomatic fatigue treatment^e^ | 0 (0.0) | 14 (3.8) | 23 (6.4) | 46 (13.3) | 60 (17.1) | 55 (27.1) |
| Sleeping aids treatment^e^ | 7 (3.6) | 27 (7.4) | 48 (13.3) | 66 (19.0) | 73 (20.8) | 55 (27.1) |
| Pain treatment^e^ | 75 (39.1) | 137 (37.6) | 164 (45.4) | 175 (50.4) | 214 (61.0) | 123 (60.6) |
| DMT |  |  |  |  |  |  |
| Dimethyl fumarate | 50 (26.0) | 87 (23.9) | 86 (23.8) | 86 (24.8) | 81 (23.1) | 34 (16.7) |
| Fingolimod | 40 (20.8) | 72 (19.8) | 59 (16.3) | 55 (15.9) | 55 (15.7) | 30 (14.8) |
| Natalizumab | 34 (17.7) | 73 (20.1) | 82 (22.7) | 80 (23.1) | 76 (21.7) | 62 (30.5) |
| Rituximab | 59 (30.7) | 106 (29.1) | 109 (30.2) | 103 (29.7) | 117 (33.3) | 64 (31.5) |
| Teriflunomide | 9 (4.7) | 26 (7.1) | 25 (6.9) | 23 (6.6) | 22 (6.3) | 13 (6.4) |
| MS duration (years) |  |  |  |  |  |  |
| 0-10 | 148 (77.1) | 267 (73.6) | 263 (73.3) | 261 (75.2) | 280 (80.0) | 164 (80.8) |
| >10 | 44 (22.9) | 96 (26.4) | 96 (26.7) | 86 (24.8) | 70 (20.0) | 39 (19.2) |
| Relapse previous year | 54 (28.1) | 108 (29.7) | 123 (34.1) | 126 (36.3) | 118 (33.6) | 95 (46.8) |
| New cerebral lesion previous year | 54 (33.1) | 91 (29.7) | 87 (29.6) | 90 (30.8) | 88 (29.1) | 61 (35.3) |
| FSMC total Score, mean (SD) | 24.8 (8.1) | 30.2 (8.0) | 44.0 (12.2) | 55.1 (9.4) | 71.2 (9.7) | 86.5 (8.1) |
| EDSS Score, median (IQR) | 1.0 (0.0-2.0) | 1.5 (0.0-2.0) | 2.0 (1.0-2.5) | 2.0 (1.0-3.0) | 2.0 (1.5-3.0) | 2.5 (1.5-3.5) |
| SDMT Score, mean (SD) | 57.0 (10.9) | 56.0 (11.2) | 53.5 (10.6) | 52.1 (11.3) | 50.4 (11.4) | 47.7 (10.7) |
| MSIS-29 Physical Score, mean (SD) | 3.3 (8.0) | 6.3 (8.8) | 12.9 (13.9) | 20.3 (15.9) | 27.9 (18.3) | 41.1 (20.1) |
| MSIS-29 Psychological Score, mean (SD) | 9.3 (14.0) | 14.3 (14.1) | 24.3 (18.3) | 33.2 (21.2) | 42.0 (21.8) | 51.9 (23.0) |
| EQ-5D VAS Score, mean (SD) | 87.5 (13.0) | 82.1 (15.4) | 77.0 (14.5) | 68.6 (18.3) | 64.8 (19.2) | 55.1 (21.7) |
| MS-symptoms inventory Score, mean (SD) | 2.0 (2.2) | 3.8 (2.7) | 6.7 (4.1) | 8.3 (3.5) | 12.1 (4.6) | 16.3 (5.6) |
| ^a^Restricted to participants 18-64 years old.  ^b^Diagnosed within 5 years prior to DMT start according to the Charlson Comorbidity Index.  ^c^Diagnosed within five years prior to DMT start.  ^d^All mental and behavioral disorders except depression and anxiety disorders.  ^e^Dispensed prescribed drugs within 1 year prior to DMT start.  DMT, disease-modifying therapy; EDSS, Expanded Disability Status Scale; EQ-5D VAS, EuroQol Visual Analogue Scale; FSMC, Fatigue Scale for Motor and Cognitive function; MS, multiple sclerosis; MSIS-29, MS Impact Scale; SDMT, Symbol Digit Modalities Test. | | | | | | |

| eTable 5 ORs of belonging to FSMC total trajectories (compared with the no FSMC total one) in a multivariable model including region of residence and excluding FSMC total, in addition to all potential predictors listed, N=1,818 participants with RRMS on *DMT switch* | | | | | |
| --- | --- | --- | --- | --- | --- |
|  | **FSMC total trajectories according to FSMC total starting values** | | | | |
|  | **Low** | **Mild** | **Moderate** | **Moderate/severe** | **Severe** |
|  | **OR (95% CI)** | **OR (95% CI)** | **OR (95% CI)** | **OR (95% CI)** | **OR (95% CI)** |
| Age, years |  |  |  |  |  |
| 18-29 | Ref. | Ref. | Ref. | Ref. | Ref. |
| 30-44 | 1.15 (0.82; 1.61) | 1.17 (0.77; 1.77) | 1.06 (0.70; 1.62) | 1.35 (0.81; 2.26) | 1.38 (0.79; 2.38) |
| >44 | 0.56 (0.37; 0.83) | 0.62 (0.40; 0.98) | 0.73 (0.46; 1.17) | 0.68 (0.40; 1.17) | 0.55 (0.31; 1.00) |
| Female versus Male | 1.40 (0.84; 2.32) | 1.85 (1.03; 3.32) | 3.00 (1.56; 5.76) | 4.50 (2.08; 9.76) | 8.34 (3.27; 21.26) |
| Born in Sweden versus born outside Sweden | 0.70 (0.29; 1.70) | 0.73 (0.27; 1.97) | 0.58 (0.21; 1.58) | 0.55 (0.18; 1.68) | 0.53 (0.15; 1.87) |
| Years of education>12 versus ≤12 | 1.31 (0.83; 2.08) | 1.00 (0.61; 1.66) | 0.87 (0.51; 1.48) | 0.93 (0.50; 1.72) | 0.82 (0.40; 1.66) |
| Disability pension previous year^a^, days | 1.00 (0.99; 1.01) | 1.00 (0.99; 1.01) | 1.00 (0.99; 1.01) | 1.00 (0.99; 1.01) | 1.00 (0.99; 1.01) |
| Sick leave previous year^a^, days | 1.02 (1.00; 1.04) | 1.03 (1.01; 1.05) | 1.03 (1.01; 1.05) | 1.03 (1.01; 1.05) | 1.03 (1.01; 1.05) |
| Comorbidity≥1^b^ versus none | 1.00 (0.39; 2.54) | 0.90 (0.33; 2.50) | 0.85 (0.30; 2.42) | 0.91 (0.28; 2.96) | 0.66 (0.19; 2.34) |
| History of depression^c^, yes versus no | 2.79 (0.26; 30.11) | 3.88 (0.35; 43.03) | 2.90 (0.26; 32.71) | 4.19 (0.34; 52.37) | 3.01 (0.22; 41.48) |
| History of anxiety^c^, yes versus no | 1.55 (0.28; 8.60) | 1.06 (0.14; 7.77) | 1.50 (0.20; 11.25) | 1.46 (0.14; 14.93) | 1.92 (0.18; 20.56) |
| History of other psychiatric comorbidities^c,d^, yes versus no | 0.37 (0.07; 1.89) | 1.42 (0.27; 7.45) | 1.77 (0.34; 9.28) | 1.41 (0.22; 8.98) | 1.71 (0.25; 11.77) |
| History of antidepressants treatment^e^, yes versus no | 0.89 (0.37; 2.16) | 1.36 (0.53; 3.50) | 1.63 (0.63; 4.20) | 1.91 (0.65; 5.62) | 2.53 (0.79; 8.13) |
| History of anxiolytics treatment^d^, yes versus no | 1.84 (0.28; 11.97) | 1.21 (0.15; 9.91) | 0.66 (0.08; 5.50) | 1.21 (0.11; 13.17) | 1.21 (0.10; 14.04) |
| History of symptomatic fatigue treatment^e^, yes versus no | n/a | n/a | n/a | n/a | n/a |
| History of sleeping aids treatment^e^, yes versus no | 1.70 (0.58; 5.01) | 1.97 (0.60; 6.46) | 2.41 (0.76; 7.70) | 1.71 (0.46; 6.33) | 1.63 (0.42; 6.33) |
| History of pain treatment^e^, yes versus no | 0.66 (0.42; 1.05) | 0.65 (0.39; 1.10) | 0.73 (0.42; 1.27) | 0.79 (0.44; 1.44) | 0.58 (0.29; 1.15) |
| DMT |  |  |  |  |  |
| Rituximab | Ref. | Ref. | Ref. | Ref. | Ref. |
| Dimethyl fumarate | 0.86 (0.54; 1.35) | 0.94 (0.57; 1.57) | 1.12 (0.66; 1.93) | 1.00 (0.53; 1.87) | 0.76 (0.36; 1.61) |
| Fingolimod | 0.92 (0.56; 1.52) | 0.81 (0.43; 1.52) | 0.79 (0.41; 1.49) | 0.79 (0.36; 1.73) | 0.73 (0.30; 1.74) |
| Natalizumab | 0.86 (0.50; 1.47) | 0.82 (0.44; 1.55) | 0.76 (0.40; 1.43) | 0.64 (0.30; 1.37) | 0.83 (0.37; 1.86) |
| Teriflunomide | 1.85 (0.80; 4.29) | 2.16 (0.80; 5.87) | 2.01 (0.71; 5.71) | 2.54 (0.73; 8.81) | 2.88 (0.73; 11.31) |
| MS duration>10 years versus ≤10 years | 1.21 (0.91; 1.61) | 1.19 (0.86; 1.65) | 1.07 (0.76; 1.51) | 0.92 (0.63; 1.35) | 0.90 (0.58; 1.40) |
| Any relapse versus none in the previous year | 1.18 (0.68; 2.05) | 1.36 (0.72; 2.57) | 1.20 (0.64; 2.25) | 1.13 (0.55; 2.33) | 1.66 (0.74; 3.72) |
| Any new cerebral lesions versus none in the previous year | 0.71 (0.42; 1.18) | 0.61 (0.32; 1.15) | 0.66 (0.34; 1.29) | 0.62 (0.28; 1.33) | 0.53 (0.23; 1.24) |
| FSMC total Score | 1.09 (1.01; 1.18) | 1.28 (1.17; 1.39) | 1.42 (1.29; 1.55) | 1.62 (1.46; 1.80) | 1.89 (1.59; 2.25) |
| EDSS Score | 1.10 (0.83; 1.45) | 1.18 (0.87; 1.60) | 1.20 (0.87; 1.66) | 1.27 (0.88; 1.84) | 1.04 (0.70; 1.54) |
| SDMT Score | 1.00 (0.97; 1.03) | 0.99 (0.97; 1.02) | 0.99 (0.96; 1.02) | 0.99 (0.95; 1.03) | 0.97 (0.93; 1.01) |
| MSIS-29 Physical Score | 0.96 (0.90; 1.04) | 0.97 (0.90; 1.04) | 0.99 (0.92; 1.06) | 0.97 (0.90; 1.05) | 1.00 (0.93; 1.08) |
| MSIS-29 Psychological Score | 1.01 (0.99; 1.04) | 1.04 (1.01; 1.07) | 1.05 (1.02; 1.08) | 1.06 (1.03; 1.09) | 1.06 (1.03; 1.09) |
| EQ-5D VAS Score | 1.00 (0.98; 1.03) | 1.01 (0.99; 1.04) | 1.00 (0.97; 1.02) | 1.02 (0.99; 1.05) | 1.02 (0.99; 1.05) |
| MS-symptoms inventory Score | 1.62 (1.27; 2.07) | 1.93 (1.54; 2.41) | 1.87 (1.42; 2.46) | 2.34 (1.82; 3.01) | 2.41 (1.81; 3.21) |
| ^a^Restricted to participants 18-64 years old.  ^b^Diagnosed within 5 years prior to DMT start according to the Charlson Comorbidity Index.  ^c^Diagnosed within five years prior to DMT start.  ^d^All mental and behavioral disorders except depression and anxiety disorders.  ^e^Dispensed prescribed drugs within 1 year prior to DMT start.  DMT, disease-modifying therapy; EDSS, Expanded Disability Status Scale; EQ-5D VAS, EuroQol Visual Analogue Scale; FSMC, Fatigue Scale for Motor and Cognitive function; MS, multiple sclerosis; MSIS-29, MS Impact Scale; SDMT, Symbol Digit Modalities Test. | | | | | |

| eTable 6 ORs of belonging to FSMC total trajectories (compared with the FSMC total one) in a multivariable model including region of residence and FSMC total, in addition to all potential predictors listed, N=1,587 participants with RRMS on *DMT switch* | | | | | |
| --- | --- | --- | --- | --- | --- |
|  | **FSMC total trajectories according to FSMC total starting values** | | | | |
|  | **Low** | **Mild** | **Moderate** | **Moderate/severe** | **Severe** |
|  | **OR (95% CI)** | **OR (95% CI)** | **OR (95% CI)** | **OR (95% CI)** | **OR (95% CI)** |
| Age, years |  |  |  |  |  |
| 18-29 | Ref. | Ref. | Ref. | Ref. | Ref. |
| 30-44 | 1.14 (0.79; 1.66) | 1.09 (0.60; 2.01) | 0.93 (0.43; 2.01) | 1.08 (0.38; 3.09) | 1.02 (0.29; 3.56) |
| >44 | 0.54 (0.35; 0.84) | 0.50 (0.23; 1.09) | 0.51 (0.19; 1.37) | 0.41 (0.12; 1.35) | 0.31 (0.07; 1.30) |
| Female versus Male | 1.35 (0.79; 2.30) | 1.46 (0.63; 3.37) | 2.12 (0.72; 6.22) | 2.78 (0.73; 10.49) | 4.90 (0.83; 29.01) |
| Born in Sweden versus born outside Sweden | 0.65 (0.23; 1.81) | 0.43 (0.09; 2.06) | 0.26 (0.04; 1.73) | 0.18 (0.02; 2.03) | 0.13 (0.01; 2.50) |
| Years of education>12 versus ≤12 | 1.39 (0.81; 2.39) | 1.31 (0.58; 2.99) | 1.35 (0.47; 3.87) | 1.70 (0.48; 6.05) | 1.66 (0.34; 8.08) |
| Disability pension previous year^a^, days | 1.00 (0.99; 1.01) | 1.00 (0.99; 1.01) | 1.00 (0.99; 1.01) | 1.00 (0.98; 1.01) | 1.00 (0.98; 1.01) |
| Sick leave previous year^a^, days | 1.02 (1.00; 1.04) | 1.03 (1.01; 1.05) | 1.03 (1.01; 1.06) | 1.04 (1.01; 1.06) | 1.04 (1.01; 1.07) |
| Comorbidity≥1^b^ versus none | 1.05 (0.36; 3.03) | 1.04 (0.23; 4.80) | 1.08 (0.15; 7.55) | 1.32 (0.11; 16.29) | 1.07 (0.06; 20.83) |
| History of depression^c^, yes versus no | 2.63 (0.19; 36.89) | 5.63 (0.29; 107.64) | 4.37 (0.16; 118.61) | 6.78 (0.13; 352.30) | 5.82 (0.06; 579.00) |
| History of anxiety^c^, yes versus no | 1.35 (0.17; 10.79) | 0.67 (0.04; 10.19) | 0.84 (0.04; 16.91) | 0.69 (0.02; 24.60) | 0.66 (0.01; 29.99) |
| History of other psychiatric comorbidities^c,d^, yes versus no | 0.25 (0.03; 1.83) | 0.65 (0.05; 8.40) | 0.60 (0.03; 10.39) | 0.32 (0.01; 9.62) | 0.27 (0.01; 14.40) |
| History of antidepressants treatment^e^, yes versus no | 0.59 (0.21; 1.69) | 0.57 (0.13; 2.50) | 0.50 (0.09; 2.82) | 0.39 (0.04; 3.70) | 0.34 (0.02; 5.77) |
| History of anxiolytics treatment^d^, yes versus no | 3.09 (0.45; 21.19) | 5.22 (0.41; 66.02) | 5.33 (0.26; 109.63) | 18.34 (0.46; 737.53) | n/a |
| History of symptomatic fatigue treatment^e^, yes versus no | n/a | n/a | n/a | n/a | n/a |
| History of sleeping aids treatment^e^, yes versus no | 2.33 (0.65; 8.28) | 5.04 (0.74; 34.47) | 8.91 (0.81; 97.90) | 9.62 (0.43; 214.03) | 15.95 (0.30; 840.85) |
| History of pain treatment^e^, yes versus no | 0.64 (0.38; 1.06) | 0.64 (0.30; 1.35) | 0.74 (0.26; 2.05) | 0.86 (0.22; 3.33) | 0.70 (0.12; 3.98) |
| DMT |  |  |  |  |  |
| Rituximab | Ref. | Ref. | Ref. | Ref. | Ref. |
| Dimethyl fumarate | 0.86 (0.51; 1.44) | 0.92 (0.42; 2.01) | 1.11 (0.38; 3.25) | 0.93 (0.20; 4.26) | 0.67 (0.10; 4.47) |
| Fingolimod | 0.87 (0.47; 1.60) | 0.65 (0.20; 2.08) | 0.54 (0.11; 2.74) | 0.47 (0.05; 4.10) | 0.39 (0.03; 5.94) |
| Natalizumab | 0.89 (0.48; 1.64) | 0.95 (0.37; 2.46) | 0.92 (0.27; 3.13) | 0.84 (0.17; 4.13) | 1.29 (0.16; 10.21) |
| Teriflunomide | 1.89 (0.69; 5.13) | 2.15 (0.44; 10.61) | 1.98 (0.25; 15.79) | 2.66 (0.18; 39.34) | 2.84 (0.10; 81.00) |
| MS duration>10 years versus ≤10 years | 1.18 (0.86; 1.63) | 1.20 (0.77; 1.87) | 1.10 (0.62; 1.95) | 0.99 (0.47; 2.07) | 1.01 (0.41; 2.52) |
| Any relapse versus none in the previous year | 1.29 (0.69; 2.42) | 1.94 (0.78; 4.80) | 2.21 (0.74; 6.62) | 2.71 (0.67; 11.01) | 5.82 (0.96; 35.21) |
| Any new cerebral lesions versus none in the previous year | 0.72 (0.41; 1.28) | 0.67 (0.27; 1.64) | 0.76 (0.25; 2.29) | 0.73 (0.16; 3.31) | 0.61 (0.09; 4.06) |
| FSMC total Score | 1.09 (1.01; 1.18) | 1.28 (1.17; 1.39) | 1.42 (1.29; 1.55) | 1.62 (1.46; 1.80) | 1.89 (1.59; 2.25) |
| EDSS Score | 1.06 (0.79; 1.41) | 1.01 (0.68; 1.50) | 0.94 (0.57; 1.56) | 0.92 (0.45; 1.86) | 0.68 (0.29; 1.61) |
| SDMT Score | 1.01 (0.98; 1.03) | 1.01 (0.97; 1.06) | 1.02 (0.95; 1.09) | 1.02 (0.94; 1.12) | 1.01 (0.91; 1.12) |
| MSIS-29 Physical Score | 0.94 (0.88; 1.01) | 0.91 (0.85; 0.99) | 0.92 (0.85; 0.99) | 0.88 (0.81; 0.96) | 0.90 (0.82; 0.98) |
| MSIS-29 Psychological Score | 1.00 (0.97; 1.03) | 0.99 (0.96; 1.03) | 0.98 (0.93; 1.02) | 0.97 (0.92; 1.02) | 0.94 (0.88; 1.00) |
| EQ-5D VAS Score | 1.01 (0.98; 1.03) | 1.03 (1.00; 1.06) | 1.02 (0.98; 1.07) | 1.06 (1.00; 1.12) | 1.06 (1.00; 1.13) |
| MS-symptoms inventory Score | 1.61 (1.25; 2.09) | 1.99 (1.54; 2.57) | 1.97 (1.47; 2.64) | 2.55 (1.84; 3.52) | 2.69 (1.81; 3.98) |
| ^a^Restricted to participants 18-64 years old.  ^b^Diagnosed within 5 years prior to DMT start according to the Charlson Comorbidity Index.  ^c^Diagnosed within five years prior to DMT start.  ^d^All mental and behavioral disorders except depression and anxiety disorders.  ^e^Dispensed prescribed drugs within 1 year prior to DMT start.  DMT, disease-modifying therapy; EDSS, Expanded Disability Status Scale; EQ-5D VAS, EuroQol Visual Analogue Scale; FSMC, Fatigue Scale for Motor and Cognitive function; MS, multiple sclerosis; MSIS-29, MS Impact Scale; SDMT, Symbol Digit Modalities Test. | | | | | |

| eTable 7 ORs of belonging to FSMC cognitive trajectories (compared with the no FSMC cognitive one) in a multivariable model including region of residence and excluding FSMC cognitive, in addition to all potential predictors listed, N=1,587 participants with RRMS on *first DMT* | | | | |
| --- | --- | --- | --- | --- |
|  | **FSMC cognitive trajectories according to FSMC cognitive starting values** | | | |
|  | **Low** | **Mild** | **Moderate** | **Severe** |
|  | **OR (95% CI)** | **OR (95% CI)** | **OR (95% CI)** | **OR (95% CI)** |
| Age, years |  |  |  |  |
| 18-29 | Ref. | Ref. | Ref. | Ref. |
| 30-44 | 0.93 (0.66; 1.29) | 1.13 (0.81; 1.55) | 1.23 (0.84; 1.81) | 1.29 (0.82; 2.03) |
| >44 | 0.92 (0.59; 1.45) | 0.83 (0.53; 1.31) | 0.75 (0.45; 1.26) | 0.86 (0.46; 1.61) |
| Female versus Male | 1.66 (1.05; 2.61) | 2.53 (1.60; 3.99) | 3.34 (1.94; 5.72) | 8.12 (3.97; 16.60) |
| Born in Sweden versus born outside Sweden | 1.18 (0.55; 2.56) | 0.94 (0.44; 2.01) | 0.99 (0.39; 2.48) | 0.79 (0.29; 2.15) |
| Years of education>12 versus ≤12 | 1.21 (0.75; 1.95) | 0.74 (0.47; 1.18) | 0.87 (0.50; 1.53) | 0.91 (0.46; 1.81) |
| Disability pension previous year^a^, days | 1.00 (0.99; 1.00) | 1.00 (0.99; 1.00) | 1.00 (0.99; 1.00) | 1.00 (0.99; 1.00) |
| Sick leave previous year^a^, days | 1.00 (0.99; 1.01) | 1.00 (1.00; 1.01) | 1.01 (1.00; 1.01) | 1.01 (1.00; 1.02) |
| Comorbidity≥1^b^ versus none | 0.66 (0.27; 1.62) | 1.20 (0.52; 2.77) | 1.04 (0.39; 2.79) | 0.56 (0.17; 1.82) |
| History of depression^c^, yes versus no | 1.97 (0.16; 24.43) | 3.54 (0.33; 38.42) | 9.97 (0.79; 126.35) | 7.89 (0.56; 111.71) |
| History of anxiety^c^, yes versus no | 10.05 (1.57; 64.18) | 4.56 (0.76; 27.23) | 7.35 (1.07; 50.42) | 10.72 (1.41; 81.56) |
| History of other psychiatric comorbidities^c,d^, yes versus no | 0.56 (0.15; 2.13) | 1.71 (0.49; 5.98) | 0.52 (0.13; 2.07) | 1.33 (0.32; 5.54) |
| History of antidepressants treatment^e^, yes versus no | 1.85 (0.58; 5.87) | 1.71 (0.55; 5.32) | 1.91 (0.54; 6.82) | 3.05 (0.77; 12.05) |
| History of anxiolytics treatment^d^, yes versus no | 1.91 (0.42; 8.77) | 1.27 (0.26; 6.14) | 0.91 (0.16; 5.15) | 0.88 (0.14; 5.58) |
| History of symptomatic fatigue treatment^e^, yes versus no | 0.21 (0.01; 5.67) | 1.23 (0.09; 17.05) | 0.36 (0.02; 7.45) | 0.76 (0.03; 17.86) |
| History of sleeping aids treatment^e^, yes versus no | 0.45 (0.17; 1.18) | 0.58 (0.22; 1.51) | 0.28 (0.09; 0.85) | 0.27 (0.09; 0.85) |
| History of pain treatment^e^, yes versus no | 0.98 (0.56; 1.72) | 0.83 (0.47; 1.46) | 1.30 (0.65; 2.60) | 1.96 (0.92; 4.19) |
| DMT |  |  |  |  |
| Rituximab | Ref. | Ref. | Ref. | Ref. |
| Dimethyl fumarate | 0.80 (0.46; 1.38) | 0.92 (0.55; 1.56) | 0.73 (0.38; 1.39) | 1.28 (0.59; 2.77) |
| Fingolimod | 1.08 (0.44; 2.69) | 1.12 (0.45; 2.78) | 0.49 (0.15; 1.61) | 0.47 (0.11; 2.01) |
| Glatiramer acetate | 0.89 (0.24; 3.37) | 1.08 (0.31; 3.71) | 2.25 (0.47; 10.91) | 1.01 (0.14; 7.32) |
| Interferon^f^ | 0.37 (0.16; 0.87) | 0.49 (0.23; 1.06) | 0.28 (0.10; 0.80) | 0.24 (0.07; 0.79) |
| Natalizumab | 1.33 (0.71; 2.46) | 1.18 (0.64; 2.17) | 1.14 (0.56; 2.34) | 0.96 (0.41; 2.28) |
| Teriflunomide | 4.17 (1.09; 15.95) | 2.29 (0.56; 9.29) | 6.45 (1.37; 30.39) | 11.55 (1.89; 70.42) |
| MS duration>10 years versus ≤10 years | 0.56 (0.28; 1.11) | 1.02 (0.56; 1.85) | 0.60 (0.27; 1.35) | 0.68 (0.26; 1.79) |
| Any relapse versus none in the previous year | 0.88 (0.53; 1.45) | 0.66 (0.40; 1.09) | 0.81 (0.46; 1.43) | 0.96 (0.50; 1.86) |
| Any new cerebral lesions versus none in the previous year | 1.15 (0.73; 1.79) | 1.20 (0.73; 1.96) | 1.03 (0.61; 1.75) | 0.65 (0.32; 1.30) |
| FSMC cognitive Score | 1.25 (1.06; 1.48) | 1.50 (1.28; 1.76) | 1.64 (1.39; 1.94) | 2.60 (1.98; 3.41) |
| EDSS Score | 0.93 (0.70; 1.22) | 1.09 (0.82; 1.44) | 0.91 (0.65; 1.27) | 0.99 (0.68; 1.45) |
| SDMT Score | 0.99 (0.97; 1.02) | 0.99 (0.97; 1.02) | 0.98 (0.96; 1.01) | 0.98 (0.95; 1.02) |
| MSIS-29 Physical Score | 0.96 (0.92; 1.00) | 0.98 (0.94; 1.02) | 0.97 (0.93; 1.01) | 0.99 (0.94; 1.04) |
| MSIS-29 Psychological Score | 1.02 (1.00; 1.04) | 1.02 (1.00; 1.04) | 1.03 (1.01; 1.05) | 1.05 (1.02; 1.08) |
| EQ-5D VAS Score | 1.03 (1.00; 1.05) | 1.00 (0.98; 1.02) | 1.01 (0.98; 1.04) | 1.01 (0.98; 1.04) |
| MS-symptoms inventory Score | 1.55 (1.23; 1.96) | 1.46 (1.13; 1.88) | 1.79 (1.43; 2.25) | 1.91 (1.43; 2.55) |
| ^a^Restricted to participants 18-64 years old.  ^b^Diagnosed within 5 years prior to DMT start according to the Charlson Comorbidity Index.  ^c^Diagnosed within five years prior to DMT start.  ^d^All mental and behavioral disorders except depression and anxiety disorders.  ^e^Dispensed prescribed drugs within 1 year prior to DMT start.  ^f^Interferon beta-1a, peginterferon beta 1 a, and interferon beta-1b.  DMT, disease-modifying therapy; EDSS, Expanded Disability Status Scale; EQ-5D VAS, EuroQol Visual Analogue Scale; FSMC, Fatigue Scale for Motor and Cognitive function; MS, Multiple Sclerosis; MSIS-29, MS Impact Scale; OR, Odds Ratio; SDMT, Symbol Digit Modalities Test. | | | | |

| eTable 8 ORs of belonging to FSMC motor trajectories (compared with the no FSMC motor one) in a multivariable model including region of residence and excluding FSMC motor, in addition to all potential predictors listed, N=1,587 participants with RRMS on *first DMT* | | | | |
| --- | --- | --- | --- | --- |
|  | **FSMC motor trajectories according to FSMC motor starting values** | | | |
|  | **Low** | **Mild** | **Moderate** | **Severe** |
|  | **OR (95% CI)** | **OR (95% CI)** | **OR (95% CI)** | **OR (95% CI)** |
| Age, years |  |  |  |  |
| 18-29 | Ref. | Ref. | Ref. | Ref. |
| 30-44 | 0.96 (0.73; 1.27) | 0.99 (0.72; 1.36) | 1.18 (0.85; 1.64) | 1.17 (0.81; 1.70) |
| >44 | 1.01 (0.66; 1.56) | 1.00 (0.62; 1.63) | 1.11 (0.68; 1.80) | 1.24 (0.72; 2.15) |
| Female versus Male | 1.06 (0.71; 1.58) | 1.81 (1.13; 2.91) | 2.15 (1.31; 3.53) | 3.95 (2.08; 7.51) |
| Born in Sweden versus born outside Sweden | 0.99 (0.51; 1.93) | 0.81 (0.39; 1.70) | 0.58 (0.27; 1.23) | 0.44 (0.19; 1.05) |
| Years of education>12 versus ≤12 | 0.93 (0.61; 1.43) | 0.83 (0.49; 1.41) | 0.61 (0.35; 1.06) | 0.67 (0.36; 1.28) |
| Disability pension previous year^a^, days | 0.99 (0.97; 1.00) | 1.00 (0.99; 1.00) | 1.00 (0.99; 1.01) | 1.00 (0.99; 1.01) |
| Sick leave previous year^a^, days | 1.00 (0.99; 1.01) | 1.01 (1.00; 1.02) | 1.01 (1.00; 1.02) | 1.01 (1.00; 1.02) |
| Comorbidity≥1^b^ versus none | 0.71 (0.31; 1.62) | 1.43 (0.56; 3.66) | 1.37 (0.51; 3.63) | 1.08 (0.36; 3.27) |
| History of depression^c^, yes versus no | 1.58 (0.15; 16.97) | 2.42 (0.21; 27.79) | 8.87 (0.80; 98.28) | 6.03 (0.49; 73.85) |
| History of anxiety^c^, yes versus no | 1.59 (0.45; 5.58) | 1.75 (0.43; 7.04) | 1.58 (0.40; 6.30) | 2.09 (0.47; 9.30) |
| History of other psychiatric comorbidities^c,d^, yes versus no | 1.55 (0.42; 5.76) | 2.05 (0.52; 8.07) | 1.11 (0.27; 4.50) | 1.97 (0.45; 8.59) |
| History of antidepressants treatment^e^, yes versus no | 1.96 (0.67; 5.72) | 1.79 (0.57; 5.63) | 2.52 (0.81; 7.86) | 2.98 (0.89; 9.99) |
| History of anxiolytics treatment^d^, yes versus no | 1.92 (0.44; 8.40) | 0.34 (0.05; 2.06) | 0.71 (0.13; 3.92) | 0.74 (0.12; 4.53) |
| History of symptomatic fatigue treatment^e^, yes versus no | n/a | n/a | n/a | n/a |
| History of sleeping aids treatment^e^, yes versus no | 0.61 (0.27; 1.40) | 0.51 (0.20; 1.31) | 0.34 (0.12; 0.93) | 0.35 (0.12; 1.05) |
| History of pain treatment^e^, yes versus no | 1.28 (0.81; 2.03) | 1.46 (0.86; 2.47) | 1.73 (1.01; 2.97) | 2.61 (1.41; 4.83) |
| DMT |  |  |  |  |
| Rituximab | Ref. | Ref. | Ref. | Ref. |
| Dimethyl fumarate | 0.88 (0.56; 1.40) | 0.93 (0.54; 1.63) | 0.91 (0.49; 1.67) | 0.86 (0.43; 1.70) |
| Fingolimod | 0.77 (0.35; 1.70) | 1.04 (0.40; 2.67) | 0.34 (0.11; 1.03) | 0.43 (0.13; 1.44) |
| Glatiramer acetate | 1.04 (0.36; 3.02) | 0.80 (0.19; 3.34) | 1.98 (0.49; 8.02) | 1.90 (0.38; 9.63) |
| Interferon^f^ | 0.51 (0.25; 1.06) | 0.42 (0.18; 0.96) | 0.48 (0.20; 1.14) | 0.36 (0.15; 0.87) |
| Natalizumab | 1.53 (0.87; 2.68) | 1.37 (0.70; 2.67) | 1.31 (0.64; 2.67) | 0.80 (0.35; 1.82) |
| Teriflunomide | 2.42 (0.82; 7.15) | 2.98 (0.82; 10.80) | 2.69 (0.70; 10.42) | 7.73 (1.90; 31.54) |
| MS duration>10 years versus ≤10 years | 1.01 (0.56; 1.82) | 0.99 (0.49; 2.00) | 1.04 (0.52; 2.10) | 0.83 (0.37; 1.87) |
| Any relapse versus none in the previous year | 0.66 (0.42; 1.05) | 0.66 (0.39; 1.12) | 0.60 (0.35; 1.06) | 0.72 (0.38; 1.37) |
| Any new cerebral lesions versus none in the previous year | 1.30 (0.88; 1.94) | 0.92 (0.56; 1.51) | 0.82 (0.49; 1.38) | 0.92 (0.51; 1.68) |
| FSMC motor Score | 1.20 (1.06; 1.36) | 1.33 (1.16; 1.52) | 1.50 (1.32; 1.70) | 1.85 (1.54; 2.23) |
| EDSS Score | 1.11 (0.89; 1.38) | 1.17 (0.91; 1.51) | 1.31 (0.99; 1.72) | 1.31 (0.94; 1.83) |
| SDMT Score | 1.00 (0.98; 1.02) | 0.99 (0.96; 1.01) | 0.98 (0.96; 1.01) | 0.98 (0.95; 1.01) |
| MSIS-29 Physical Score | 0.98 (0.94; 1.02) | 0.98 (0.94; 1.02) | 1.00 (0.96; 1.04) | 1.04 (0.99; 1.09) |
| MSIS-29 Psychological Score | 1.02 (1.00; 1.04) | 1.02 (1.00; 1.05) | 1.04 (1.01; 1.06) | 1.04 (1.02; 1.07) |
| EQ-5D VAS Score | 1.01 (0.99; 1.03) | 1.00 (0.98; 1.02) | 1.00 (0.98; 1.03) | 1.01 (0.98; 1.03) |
| MS-symptoms inventory Score | 1.30 (1.05; 1.61) | 1.53 (1.25; 1.86) | 1.50 (1.22; 1.84) | 1.52 (1.22; 1.90) |
| ^a^Restricted to participants 18-64 years old.  ^b^Diagnosed within 5 years prior to DMT start according to the Charlson Comorbidity Index.  ^c^Diagnosed within five years prior to DMT start.  ^d^All mental and behavioral disorders except depression and anxiety disorders.  ^e^Dispensed prescribed drugs within 1 year prior to DMT start.  ^f^Interferon beta-1a, peginterferon beta 1 a, and interferon beta-1b.  DMT, disease-modifying therapy; EDSS, Expanded Disability Status Scale; EQ-5D VAS, EuroQol Visual Analogue Scale; FSMC, Fatigue Scale for Motor and Cognitive function; MS, Multiple Sclerosis; MSIS-29, MS Impact Scale; OR, Odds Ratio; SDMT, Symbol Digit Modalities Test. | | | | |

| eTable 9 ORs of belonging to FSMC cognitive trajectories (compared with the no FSMC cognitive one) in a multivariable model including region of residence and excluding FSMC cognitive, in addition to all potential predictors listed, N=1,818 participants with RRMS on *DMT switch* | | | | | | | | |
| --- | --- | --- | --- | --- | --- | --- | --- | --- |
|  | **FSMC cognitive trajectories according to FSMC cognitive starting values** | | | | | | | |
|  | **Low** | | **Mild** | | **Moderate** | | **Severe** | |
|  | **OR (95% CI)** | | **OR (95% CI)** | | **OR (95% CI)** | | **OR (95% CI)** | |
| Age, years |  | |  | |  | |  | |
| 18-29 | Ref. | | Ref. | | Ref. | | Ref. | |
| 30-44 | 1.05 (0.80; 1.37) | | 1.11 (0.81; 1.52) | | 1.15 (0.80; 1.66) | | 1.34 (0.90; 1.99) | |
| >44 | 0.82 (0.59; 1.14) | | 0.71 (0.48; 1.05) | | 0.85 (0.53; 1.36) | | 0.66 (0.39; 1.11) | |
| Female versus Male | 1.56 (1.01; 2.42) | | 2.49 (1.51; 4.11) | | 3.55 (1.97; 6.40) | | 7.85 (3.86; 15.98) | |
| Born in Sweden versus born outside Sweden | 1.17 (0.56; 2.42) | | 1.09 (0.45; 2.63) | | 1.18 (0.44; 3.19) | | 1.24 (0.39; 3.92) | |
| Years of education>12 versus ≤12 | 1.20 (0.77; 1.88) | | 0.89 (0.54; 1.48) | | 0.95 (0.53; 1.71) | | 0.79 (0.42; 1.50) | |
| Disability pension previous year^a^, days | 1.00 (0.99; 1.01) | | 1.00 (0.99; 1.01) | | 1.00 (0.99; 1.01) | | 1.00 (0.99; 1.01) | |
| Sick leave previous year^a^, days | 1.01 (1.00; 1.03) | | 1.02 (1.00; 1.03) | | 1.02 (1.01; 1.03) | | 1.02 (1.01; 1.03) | |
| Comorbidity≥1^b^ versus none | 1.23 (0.54; 2.78) | | 0.98 (0.38; 2.51) | | 0.98 (0.33; 2.87) | | 0.91 (0.30; 2.79) | |
| History of depression^c^, yes versus no | 2.75 (0.28; 27.09) | | 3.80 (0.36; 39.77) | | 3.72 (0.31; 44.41) | | 3.70 (0.31; 44.82) | |
| History of anxiety^c^, yes versus no | 1.63 (0.40; 6.63) | | 1.00 (0.20; 5.00) | | 1.42 (0.23; 8.76) | | 1.46 (0.23; 9.13) | |
| History of other psychiatric comorbidities^c,d^, yes versus no | 1.00 (0.26; 3.86) | | 1.95 (0.47; 8.02) | | 1.66 (0.35; 7.97) | | 1.99 (0.41; 9.75) | |
| History of antidepressants treatment^e^, yes versus no | 1.04 (0.49; 2.22) | | 1.63 (0.72; 3.65) | | 1.89 (0.77; 4.62) | | 2.75 (1.08; 6.98) | |
| History of anxiolytics treatment^d^, yes versus no | 2.03 (0.31; 13.17) | | 1.28 (0.17; 9.48) | | 1.89 (0.20; 17.57) | | 1.80 (0.18; 17.94) | |
| History of symptomatic fatigue treatment^e^, yes versus no | 5.48 (1.13; 26.58) | | 6.21 (1.18; 32.73) | | 14.89 (2.48; 89.31) | | 19.89 (3.27; 121.15) | |
| History of sleeping aids treatment^e^, yes versus no | 1.43 (0.64; 3.20) | | 1.63 (0.67; 3.96) | | 1.44 (0.54; 3.82) | | 1.02 (0.36; 2.89) | |
| History of pain treatment^e^, yes versus no | 0.83 (0.54; 1.25) | | 0.90 (0.56; 1.45) | | 1.03 (0.57; 1.87) | | 0.94 (0.48; 1.83) | |
| DMT |  |  | |  | |  | |  |
| Rituximab | Ref. | | Ref. | | Ref. | | Ref. | |
| Dimethyl fumarate | 0.82 (0.54; 1.25) | | 0.89 (0.53; 1.47) | | 0.94 (0.49; 1.82) | | 0.78 (0.39; 1.53) | |
| Fingolimod | 0.97 (0.61; 1.52) | | 0.95 (0.53; 1.70) | | 0.89 (0.45; 1.76) | | 0.79 (0.37; 1.68) | |
| Natalizumab | 0.88 (0.55; 1.40) | | 0.89 (0.52; 1.55) | | 0.73 (0.37; 1.43) | | 0.80 (0.40; 1.62) | |
| Teriflunomide | 1.56 (0.79; 3.07) | | 1.78 (0.82; 3.86) | | 2.13 (0.83; 5.46) | | 2.59 (0.99; 6.82) | |
| MS duration>10 years versus ≤10 years | 1.15 (0.92; 1.44) | | 1.13 (0.87; 1.47) | | 0.92 (0.66; 1.27) | | 0.87 (0.61; 1.25) | |
| Any relapse versus none in the previous year | 1.08 (0.68; 1.72) | | 0.94 (0.54; 1.62) | | 0.99 (0.52; 1.88) | | 1.44 (0.72; 2.89) | |
| Any new cerebral lesions versus none in the previous year | 1.03 (0.64; 1.67) | | 0.93 (0.50; 1.71) | | 0.95 (0.48; 1.90) | | 0.64 (0.30; 1.35) | |
| FSMC cognitive Score | 1.28 (1.12; 1.46) | | 1.65 (1.40; 1.95) | | 1.94 (1.64; 2.29) | | 2.51 (2.02; 3.11) | |
| EDSS Score | 0.99 (0.78; 1.26) | | 1.06 (0.80; 1.40) | | 0.97 (0.71; 1.33) | | 0.83 (0.60; 1.17) | |
| SDMT Score | 1.00 (0.98; 1.02) | | 0.99 (0.96; 1.02) | | 1.00 (0.96; 1.03) | | 0.97 (0.94; 1.01) | |
| MSIS-29 Physical Score | 0.97 (0.92; 1.02) | | 0.96 (0.91; 1.01) | | 0.95 (0.90; 1.00) | | 0.98 (0.92; 1.04) | |
| MSIS-29 Psychological Score | 1.03 (1.01; 1.06) | | 1.06 (1.03; 1.08) | | 1.06 (1.03; 1.09) | | 1.07 (1.04; 1.11) | |
| EQ-5D VAS Score | 1.00 (0.98; 1.02) | | 1.00 (0.98; 1.02) | | 1.00 (0.97; 1.03) | | 1.01 (0.98; 1.04) | |
| MS-symptoms inventory Score | 1.42 (1.12; 1.81) | | 1.59 (1.21; 2.10) | | 1.89 (1.47; 2.44) | | 1.90 (1.38; 2.62) | |
| ^a^Restricted to participants 18-64 years old.  ^b^Diagnosed within 5 years prior to DMT start according to the Charlson Comorbidity Index.  ^c^Diagnosed within five years prior to DMT start.  ^d^All mental and behavioral disorders except depression and anxiety disorders.  ^e^Dispensed prescribed drugs within 1 year prior to DMT start.  DMT, disease-modifying therapy; EDSS, Expanded Disability Status Scale; EQ-5D VAS, EuroQol Visual Analogue Scale; FSMC, Fatigue Scale for Motor and Cognitive function; MS, multiple sclerosis; MSIS-29, MS Impact Scale; SDMT, Symbol Digit Modalities Test. | | | | | | | | |

| eTable 10 ORs of belonging to FSMC motor trajectories (compared with the no FSMC motor one) in a multivariable model including region of residence and excluding FSMC motor, in addition to all potential predictors listed, N=1,818 participants with RRMS on *DMT switch* | | | | | | | | |
| --- | --- | --- | --- | --- | --- | --- | --- | --- |
|  | **FSMC motor trajectories according to FSMC motor starting values** | | | | | | | |
|  | **Low** | | **Mild** | | **Moderate** | | **Severe** | |
|  | **OR (95% CI)** | | **OR (95% CI)** | | **OR (95% CI)** | | **OR (95% CI)** | |
| Age, years |  | |  | |  | |  | |
| 18-29 | Ref. | | Ref. | | Ref. | | Ref. | |
| 30-44 | 1.13 (0.84; 1.52) | | 0.98 (0.69; 1.38) | | 1.17 (0.76; 1.78) | | 1.32 (0.83; 2.10) | |
| >44 | 0.59 (0.41; 0.85) | | 0.72 (0.49; 1.07) | | 0.72 (0.44; 1.17) | | 0.53 (0.29; 0.94) | |
| Female versus Male | 1.58 (0.97; 2.58) | | 2.18 (1.22; 3.89) | | 3.11 (1.54; 6.28) | | 5.94 (2.55; 13.84) | |
| Born in Sweden versus born outside Sweden | 0.56 (0.25; 1.26) | | 0.59 (0.23; 1.48) | | 0.54 (0.19; 1.54) | | 0.62 (0.19; 2.02) | |
| Years of education>12 versus ≤12 | 1.10 (0.68; 1.79) | | 0.88 (0.50; 1.55) | | 0.84 (0.43; 1.65) | | 0.76 (0.33; 1.73) | |
| Disability pension previous year^a^, days | 1.00 (0.99; 1.01) | | 1.00 (1.00; 1.01) | | 1.00 (0.99; 1.01) | | 1.00 (0.99; 1.01) | |
| Sick leave previous year^a^, days | 1.01 (1.00; 1.02) | | 1.02 (1.01; 1.03) | | 1.02 (1.01; 1.03) | | 1.02 (1.01; 1.04) | |
| Comorbidity≥1^b^ versus none | 1.36 (0.59; 3.16) | | 1.53 (0.62; 3.80) | | 1.49 (0.52; 4.25) | | 1.07 (0.34; 3.40) | |
| History of depression^c^, yes versus no | 3.00 (0.50; 18.04) | | 2.60 (0.38; 18.00) | | 2.89 (0.35; 24.12) | | 2.55 (0.27; 24.00) | |
| History of anxiety^c^, yes versus no | 1.06 (0.29; 3.81) | | 0.95 (0.22; 4.19) | | 1.07 (0.18; 6.24) | | 1.31 (0.19; 9.26) | |
| History of other psychiatric comorbidities^c,d^, yes versus no | 1.44 (0.35; 5.95) | | 3.10 (0.68; 14.08) | | 2.13 (0.38; 12.09) | | 2.78 (0.44; 17.69) | |
| History of antidepressants treatment^e^, yes versus no | 1.08 (0.53; 2.19) | | 1.38 (0.64; 2.98) | | 2.07 (0.88; 4.82) | | 2.08 (0.81; 5.37) | |
| History of anxiolytics treatment^d^, yes versus no | 0.95 (0.21; 4.30) | | 0.41 (0.07; 2.37) | | 0.52 (0.07; 4.08) | | 0.70 (0.08; 6.15) | |
| History of symptomatic fatigue treatment^e^, yes versus no | 5.79 (1.07; 31.19) | | 10.71 (1.94; 59.00) | | 16.75 (2.63; 106.77) | | 24.30 (3.58; 165.09) | |
| History of sleeping aids treatment^e^, yes versus no | 0.93 (0.39; 2.22) | | 1.60 (0.64; 4.02) | | 0.98 (0.33; 2.92) | | 0.87 (0.26; 2.86) | |
| History of pain treatment^e^, yes versus no | 1.12 (0.75; 1.67) | | 1.02 (0.65; 1.61) | | 1.48 (0.86; 2.53) | | 1.08 (0.58; 2.02) | |
| DMT |  |  | |  | |  | |  |
| Rituximab | Ref. | | Ref. | | Ref. | | Ref. | |
| Dimethyl fumarate | 0.97 (0.61; 1.52) | | 1.02 (0.61; 1.72) | | 0.93 (0.46; 1.88) | | 0.74 (0.33; 1.67) | |
| Fingolimod | 0.89 (0.54; 1.47) | | 0.94 (0.52; 1.71) | | 0.97 (0.46; 2.06) | | 0.88 (0.37; 2.12) | |
| Natalizumab | 0.93 (0.55; 1.58) | | 1.06 (0.59; 1.91) | | 0.74 (0.35; 1.59) | | 0.94 (0.38; 2.30) | |
| Teriflunomide | 1.31 (0.65; 2.64) | | 1.61 (0.71; 3.65) | | 1.95 (0.69; 5.51) | | 2.63 (0.80; 8.65) | |
| MS duration>10 years versus ≤10 years | 1.17 (0.90; 1.51) | | 1.17 (0.88; 1.56) | | 0.93 (0.65; 1.32) | | 0.94 (0.62; 1.41) | |
| Any relapse versus none in the previous year | 1.02 (0.60; 1.73) | | 1.15 (0.64; 2.06) | | 1.15 (0.58; 2.31) | | 1.19 (0.54; 2.62) | |
| Any new cerebral lesions versus none in the previous year | 0.59 (0.36; 0.96) | | 0.72 (0.42; 1.22) | | 0.55 (0.28; 1.08) | | 0.54 (0.24; 1.23) | |
| FSMC motor Score | 1.31 (1.14; 1.50) | | 1.55 (1.31; 1.84) | | 1.89 (1.63; 2.19) | | 2.21 (1.80; 2.72) | |
| EDSS Score | 1.26 (0.97; 1.65) | | 1.38 (1.02; 1.88) | | 1.29 (0.88; 1.88) | | 1.17 (0.77; 1.77) | |
| SDMT Score | 1.00 (0.98; 1.03) | | 1.00 (0.98; 1.03) | | 1.00 (0.97; 1.03) | | 0.98 (0.95; 1.02) | |
| MSIS-29 Physical Score | 1.00 (0.95; 1.05) | | 1.03 (0.98; 1.09) | | 1.02 (0.96; 1.07) | | 1.05 (0.99; 1.11) | |
| MSIS-29 Psychological Score | 1.02 (0.99; 1.04) | | 1.03 (1.00; 1.05) | | 1.04 (1.00; 1.07) | | 1.03 (1.00; 1.07) | |
| EQ-5D VAS Score | 1.00 (0.98; 1.02) | | 1.00 (0.98; 1.02) | | 1.01 (0.98; 1.04) | | 1.01 (0.98; 1.04) | |
| MS-symptoms inventory Score | 1.54 (1.11; 2.14) | | 1.67 (1.19; 2.36) | | 2.08 (1.49; 2.89) | | 2.26 (1.56; 3.26) | |
| ^a^Restricted to participants 18-64 years old.  ^b^Diagnosed within 5 years prior to DMT start according to the Charlson Comorbidity Index.  ^c^Diagnosed within five years prior to DMT start.  ^d^All mental and behavioral disorders except depression and anxiety disorders.  ^e^Dispensed prescribed drugs within 1 year prior to DMT start.  DMT, disease-modifying therapy; EDSS, Expanded Disability Status Scale; EQ-5D VAS, EuroQol Visual Analogue Scale; FSMC, Fatigue Scale for Motor and Cognitive function; MS, multiple sclerosis; MSIS-29, MS Impact Scale; SDMT, Symbol Digit Modalities Test. | | | | | | | | |

| eTable 11 ORs of belonging to fatigue trajectories (compared with the no fatigue one) in a multivariable model including region of residence and excluding FSMC total, in addition to all potential predictors listed. Sensitivity analysis of participants with RRMS on *first DMT* whose trajectory assignment probability was ≥ 0.80 (N=1,319) | | | | |
| --- | --- | --- | --- | --- |
|  | **FSMC total trajectories according to FSMC total starting values** | | | |
|  | **Low** | **Mild** | **Moderate** | **Severe** |
|  | **OR (95% CI)** | **OR (95% CI)** | **OR (95% CI)** | **OR (95% CI)** |
| Age, years |  |  |  |  |
| 18-29 | Ref. | Ref. | Ref. | Ref. |
| 30-44 | 0.89 (0.66; 1.20) | 1.10 (0.80; 1.51) | 1.33 (0.92; 1.93) | 1.24 (0.81; 1.91) |
| >44 | 0.97 (0.62; 1.53) | 0.95 (0.58; 1.54) | 0.84 (0.47; 1.48) | 0.97 (0.52; 1.79) |
| Female versus Male | 1.79 (1.13; 2.86) | 2.47 (1.46; 4.19) | 3.25 (1.72; 6.14) | 7.68 (3.52; 16.74) |
| Born in Sweden versus born outside Sweden | 0.99 (0.49; 2.01) | 0.84 (0.38; 1.84) | 0.83 (0.33; 2.12) | 0.54 (0.19; 1.51) |
| Years of education>12 versus ≤12 | 1.32 (0.80; 2.16) | 1.01 (0.58; 1.77) | 0.92 (0.45; 1.88) | 0.95 (0.42; 2.18) |
| Disability pension previous year^a^, days | 0.99 (0.98; 1.00) | 1.00 (0.99; 1.00) | 1.00 (0.99; 1.01) | 1.00 (0.99; 1.01) |
| Sick leave previous year^a^, days | 1.00 (0.99; 1.01) | 1.00 (0.99; 1.01) | 1.01 (1.00; 1.02) | 1.01 (1.00; 1.02) |
| Comorbidity≥1^b^ versus none | 0.98 (0.42; 2.33) | 1.71 (0.73; 4.01) | 1.48 (0.54; 4.06) | 0.90 (0.30; 2.73) |
| History of depression^c^, yes versus no | 2.42 (0.20; 29.09) | 4.13 (0.34; 49.94) | 11.86 (0.89; 158.47) | 7.33 (0.52; 104.23) |
| History of anxiety^c^, yes versus no | 4.05 (0.83; 19.76) | 2.85 (0.54; 14.99) | 4.40 (0.80; 24.32) | 6.25 (1.05; 37.19) |
| History of other psychiatric comorbidities^c,d^, yes versus no | 1.11 (0.26; 4.67) | 1.30 (0.30; 5.64) | 0.74 (0.13; 4.03) | 1.79 (0.30; 10.74) |
| History of antidepressants treatment^e^, yes versus no | 1.38 (0.46; 4.14) | 1.86 (0.60; 5.77) | 2.03 (0.60; 6.81) | 2.64 (0.72; 9.66) |
| History of anxiolytics treatment^d^, yes versus no | 1.35 (0.32; 5.64) | 0.29 (0.06; 1.53) | 0.62 (0.12; 3.22) | 0.37 (0.06; 2.19) |
| History of symptomatic fatigue treatment^e^, yes versus no | 0.50 (0.02; 11.67) | 0.84 (0.04; 15.59) | 0.19 (0.01; 6.23) | 0.46 (0.02; 12.78) |
| History of sleeping aids treatment^e^, yes versus no | 0.61 (0.26; 1.46) | 0.53 (0.20; 1.42) | 0.28 (0.10; 0.82) | 0.37 (0.12; 1.15) |
| History of pain treatment^e^, yes versus no | 0.93 (0.55; 1.56) | 1.09 (0.63; 1.87) | 1.38 (0.73; 2.60) | 2.22 (1.07; 4.62) |
| DMT |  |  |  |  |
| Rituximab | Ref. | Ref. | Ref. | Ref. |
| Dimethyl fumarate | 0.76 (0.45; 1.29) | 0.94 (0.52; 1.68) | 0.77 (0.38; 1.57) | 0.93 (0.44; 1.98) |
| Fingolimod | 0.83 (0.33; 2.09) | 0.94 (0.34; 2.57) | 0.35 (0.09; 1.39) | 0.49 (0.11; 2.24) |
| Glatiramer acetate | 1.16 (0.32; 4.14) | 0.75 (0.18; 3.20) | 2.99 (0.54; 16.68) | 1.45 (0.20; 10.30) |
| Interferon^f^ | 0.34 (0.15; 0.77) | 0.49 (0.20; 1.23) | 0.27 (0.08; 0.97) | 0.22 (0.06; 0.84) |
| Natalizumab | 1.63 (0.81; 3.28) | 1.49 (0.70; 3.17) | 1.28 (0.51; 3.21) | 1.09 (0.41; 2.88) |
| Teriflunomide | 4.08 (1.13; 14.77) | 3.04 (0.72; 12.79) | 4.59 (0.93; 22.70) | 10.05 (1.63; 61.83) |
| MS duration>10 years versus ≤10 years | 0.69 (0.34; 1.37) | 1.10 (0.56; 2.16) | 0.76 (0.33; 1.72) | 0.65 (0.25; 1.72) |
| Any relapse versus none in the previous year | 0.78 (0.47; 1.29) | 0.71 (0.42; 1.20) | 0.79 (0.42; 1.48) | 0.95 (0.46; 1.95) |
| Any new cerebral lesions versus none in the previous year | 1.19 (0.72; 1.97) | 1.01 (0.58; 1.76) | 1.01 (0.49; 2.06) | 0.81 (0.37; 1.76) |
| FSMC total Score | 1.16 (1.08; 1.24) | 1.26 (1.19; 1.35) | 1.32 (1.21; 1.43) | 1.49 (1.37; 1.64) |
| EDSS Score | 1.06 (0.81; 1.37) | 1.27 (0.99; 1.65) | 1.13 (0.81; 1.58) | 1.29 (0.86; 1.94) |
| SDMT Score | 0.99 (0.96; 1.01) | 0.98 (0.95; 1.00) | 0.96 (0.93; 1.00) | 0.96 (0.92; 0.99) |
| MSIS-29 Physical Score | 0.96 (0.93; 1.00) | 0.98 (0.94; 1.02) | 0.97 (0.92; 1.01) | 1.00 (0.96; 1.05) |
| MSIS-29 Psychological Score | 1.01 (0.99; 1.03) | 1.02 (1.00; 1.04) | 1.02 (1.00; 1.05) | 1.04 (1.01; 1.06) |
| EQ-5D VAS Score | 1.01 (0.99; 1.04) | 1.00 (0.97; 1.03) | 1.00 (0.97; 1.03) | 1.00 (0.97; 1.03) |
| MS-symptoms inventory Score | 1.47 (1.19; 1.80) | 1.46 (1.17; 1.83) | 1.80 (1.42; 2.29) | 1.80 (1.36; 2.37) |
| ^a^Restricted to participants 18-64 years old.  ^b^Diagnosed within 5 years prior to DMT start according to the Charlson Comorbidity Index.  ^c^Diagnosed within five years prior to DMT start.  ^d^All mental and behavioral disorders except depression and anxiety disorders.  ^e^Dispensed prescribed drugs within 1 year prior to DMT start.  ^f^Interferon beta-1a, peginterferon beta 1 a, and interferon beta-1b.  DMT, disease-modifying therapy; EDSS, Expanded Disability Status Scale; EQ-5D VAS, EuroQol Visual Analogue Scale; FSMC, Fatigue Scale for Motor and Cognitive function; MS, Multiple Sclerosis; MSIS-29, MS Impact Scale; OR, Odds Ratio; SDMT, Symbol Digit Modalities Test. | | | | |

| eTable 12 ORs of belonging to fatigue trajectories (compared with the no fatigue one) in a multivariable model including region of residence and excluding FSMC total, in addition to all potential predictors listed. Sensitivity analysis of participants with RRMS on *DMT switch* whose trajectory assignment probability was ≥ 0.80 (N=1,470) | | | | | |
| --- | --- | --- | --- | --- | --- |
|  | **FSMC total trajectories according to FSMC total starting values** | | | | |
|  | **Low** | **Mild** | **Moderate** | **Moderate/severe** | **Severe** |
|  | **OR (95% CI)** | **OR (95% CI)** | **OR (95% CI)** | **OR (95% CI)** | **OR (95% CI)** |
| Age, years |  |  |  |  |  |
| 18-29 | Ref. | Ref. | Ref. | Ref. | Ref. |
| 30-44 | 1.14 (0.82; 1.57) | 1.17 (0.81; 1.68) | 1.03 (0.71; 1.49) | 1.29 (0.83; 2.01) | 1.35 (0.83; 2.22) |
| >44 | 0.58 (0.36; 0.95) | 0.72 (0.41; 1.26) | 0.87 (0.50; 1.50) | 0.77 (0.39; 1.52) | 0.72 (0.34; 1.49) |
| Female versus Male | 1.45 (0.86; 2.46) | 1.76 (0.94; 3.30) | 2.61 (1.34; 5.09) | 3.58 (1.56; 8.25) | 7.31 (2.79; 19.11) |
| Born in Sweden versus born outside Sweden | 0.54 (0.15; 1.89) | 0.54 (0.14; 2.12) | 0.39 (0.11; 1.46) | 0.53 (0.12; 2.40) | 0.46 (0.10; 2.12) |
| Years of education>12 versus ≤12 | 1.43 (0.85; 2.41) | 1.04 (0.58; 1.85) | 0.92 (0.51; 1.68) | 0.91 (0.46; 1.80) | 0.72 (0.33; 1.57) |
| Disability pension previous year^a^, days | 1.00 (0.99; 1.00) | 1.00 (0.99; 1.00) | 1.00 (0.99; 1.01) | 1.00 (0.99; 1.01) | 1.00 (0.99; 1.01) |
| Sick leave previous year^a^, days | 1.02 (1.00; 1.04) | 1.03 (1.00; 1.05) | 1.03 (1.01; 1.05) | 1.03 (1.01; 1.06) | 1.03 (1.01; 1.06) |
| Comorbidity≥1^b^ versus none | 1.02 (0.34; 3.11) | 1.09 (0.33; 3.65) | 1.05 (0.31; 3.56) | 0.91 (0.22; 3.80) | 0.76 (0.18; 3.31) |
| History of depression^c^, yes versus no | n/a | n/a | n/a | n/a | n/a |
| History of anxiety^c^, yes versus no | 1.69 (0.21; 13.85) | 2.01 (0.20; 20.41) | 2.16 (0.22; 21.36) | 2.31 (0.18; 28.96) | 3.87 (0.29; 52.10) |
| History of other psychiatric comorbidities^c,d^, yes versus no | 0.70 (0.09; 5.31) | 2.43 (0.32; 18.22) | 2.46 (0.32; 19.01) | 1.48 (0.17; 13.16) | 1.73 (0.18; 16.73) |
| History of antidepressants treatment^e^, yes versus no | 0.98 (0.34; 2.84) | 1.64 (0.51; 5.34) | 2.19 (0.68; 7.07) | 2.42 (0.60; 9.77) | 2.99 (0.69; 12.99) |
| History of anxiolytics treatment^d^, yes versus no | 1.31 (0.15; 11.43) | 0.43 (0.03; 5.35) | 0.40 (0.03; 4.90) | 0.68 (0.04; 12.46) | 0.62 (0.03; 11.92) |
| History of symptomatic fatigue treatment^e^, yes versus no | n/a | n/a | n/a | n/a | n/a |
| History of sleeping aids treatment^e^, yes versus no | 1.44 (0.49; 4.21) | 1.32 (0.42; 4.15) | 1.65 (0.52; 5.22) | 1.30 (0.37; 4.59) | 1.34 (0.34; 5.28) |
| History of pain treatment^e^, yes versus no | 0.71 (0.40; 1.26) | 0.76 (0.38; 1.50) | 0.76 (0.39; 1.51) | 0.99 (0.45; 2.21) | 0.80 (0.33; 1.90) |
| DMT |  |  |  |  |  |
| Rituximab | Ref. | Ref. | Ref. | Ref. | Ref. |
| Dimethyl fumarate | 0.89 (0.53; 1.51) | 1.04 (0.57; 1.89) | 1.29 (0.69; 2.43) | 1.07 (0.52; 2.19) | 0.85 (0.38; 1.93) |
| Fingolimod | 0.99 (0.57; 1.70) | 0.72 (0.37; 1.38) | 0.79 (0.40; 1.56) | 0.69 (0.29; 1.66) | 0.52 (0.20; 1.35) |
| Natalizumab | 0.78 (0.45; 1.38) | 0.76 (0.38; 1.51) | 0.66 (0.33; 1.30) | 0.51 (0.21; 1.19) | 0.68 (0.27; 1.73) |
| Teriflunomide | 1.63 (0.70; 3.81) | 2.27 (0.86; 5.98) | 1.68 (0.60; 4.67) | 2.72 (0.86; 8.60) | 3.01 (0.84; 10.80) |
| MS duration>10 years versus ≤10 years | 1.17 (0.86; 1.60) | 1.20 (0.83; 1.73) | 1.12 (0.77; 1.63) | 0.91 (0.59; 1.42) | 0.99 (0.60; 1.64) |
| Any relapse versus none in the previous year | 1.08 (0.57; 2.03) | 1.45 (0.68; 3.09) | 1.28 (0.59; 2.78) | 1.00 (0.40; 2.51) | 1.68 (0.61; 4.62) |
| Any new cerebral lesions versus none in the previous year | 0.65 (0.36; 1.18) | 0.56 (0.25; 1.23) | 0.69 (0.31; 1.55) | 0.56 (0.22; 1.46) | 0.50 (0.18; 1.41) |
| FSMC total Score | 1.09 (0.98; 1.21) | 1.29 (1.15; 1.44) | 1.42 (1.25; 1.61) | 1.64 (1.44; 1.86) | 1.95 (1.63; 2.33) |
| EDSS Score | 1.09 (0.80; 1.49) | 1.07 (0.76; 1.51) | 1.06 (0.75; 1.50) | 1.14 (0.75; 1.73) | 0.88 (0.57; 1.37) |
| SDMT Score | 1.00 (0.97; 1.03) | 0.99 (0.96; 1.02) | 0.99 (0.96; 1.01) | 0.99 (0.96; 1.03) | 0.97 (0.93; 1.01) |
| MSIS-29 Physical Score | 0.97 (0.91; 1.05) | 0.98 (0.91; 1.06) | 1.01 (0.93; 1.09) | 0.99 (0.91; 1.08) | 1.03 (0.94; 1.13) |
| MSIS-29 Psychological Score | 1.03 (1.00; 1.06) | 1.06 (1.02; 1.09) | 1.06 (1.03; 1.10) | 1.07 (1.03; 1.11) | 1.06 (1.02; 1.11) |
| EQ-5D VAS Score | 0.99 (0.96; 1.02) | 1.00 (0.97; 1.03) | 0.98 (0.95; 1.02) | 1.01 (0.97; 1.04) | 1.00 (0.96; 1.03) |
| MS-symptoms inventory Score | 1.56 (1.06; 2.30) | 1.83 (1.29; 2.58) | 1.76 (1.22; 2.53) | 2.22 (1.53; 3.22) | 2.20 (1.45; 3.35) |
| ^a^Restricted to participants 18-64 years old.  ^b^Diagnosed within 5 years prior to DMT start according to the Charlson Comorbidity Index.  ^c^Diagnosed within five years prior to DMT start.  ^d^All mental and behavioral disorders except depression and anxiety disorders.  ^e^Dispensed prescribed drugs within 1 year prior to DMT start.  DMT, disease-modifying therapy; EDSS, Expanded Disability Status Scale; EQ-5D VAS, EuroQol Visual Analogue Scale; FSMC, Fatigue Scale for Motor and Cognitive function; MS, multiple sclerosis; MSIS-29, MS Impact Scale; SDMT, Symbol Digit Modalities Test. | | | | | |

## Figures

**
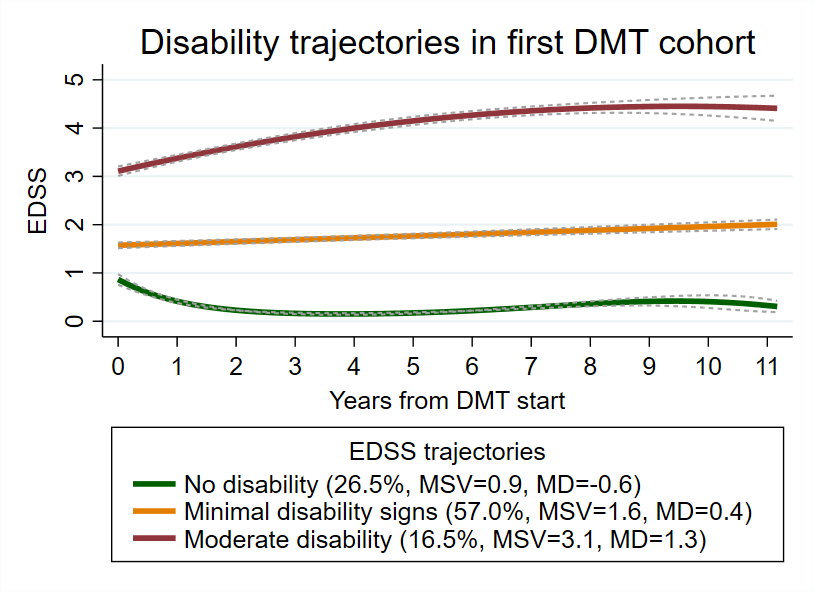
**

**
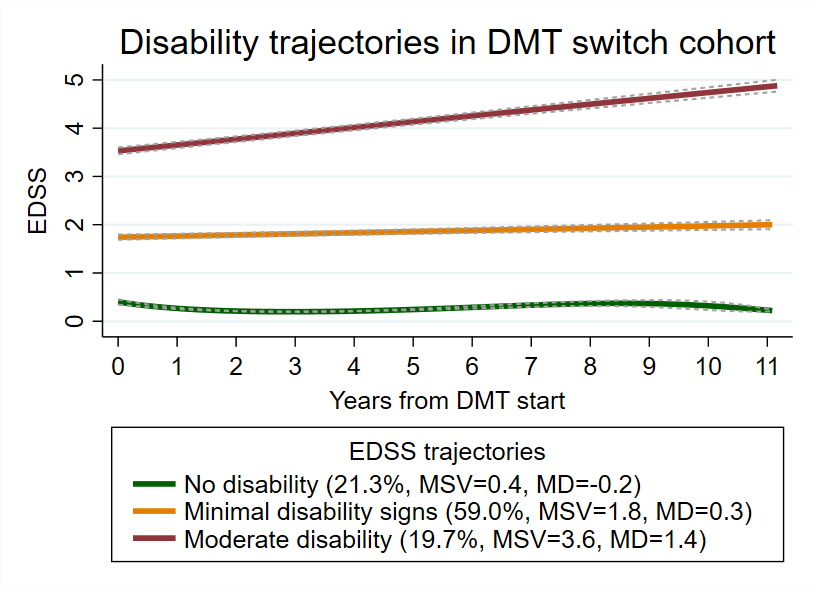
**

eFigure 1**.** Trajectories of physical disability (Expanded Disability Status Scale (EDSS)) and corresponding 95% CIs (dotted lines), over 11 years following first (upper panel) and switch (lower panel) disease-modifying therapy (DMT) initiation. Trajectory group legends describe the proportion of participants in each group relative to the entire study population, mean EDSS starting values (MSV) and the difference in EDSS scores compared to baseline (MD).


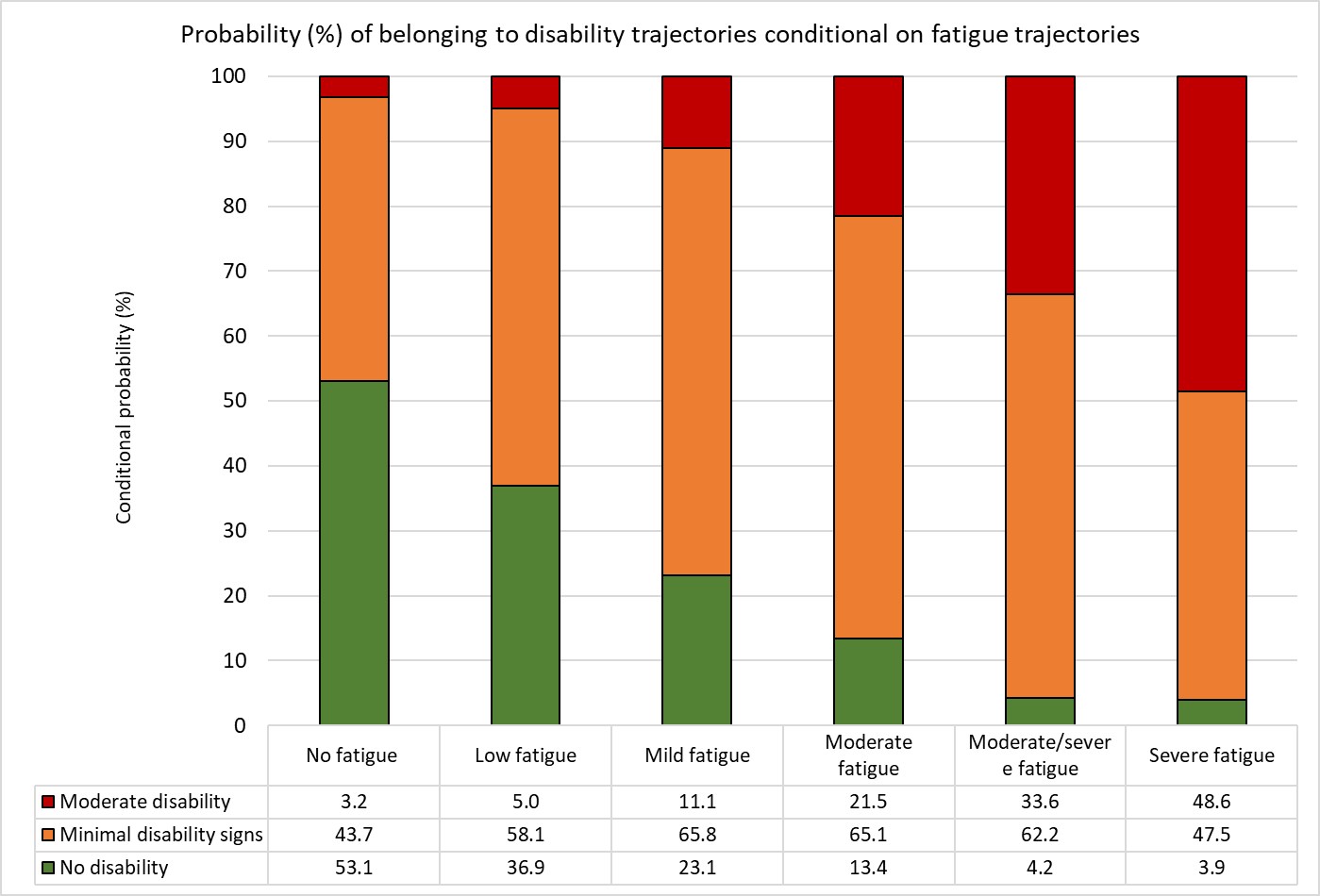


eFigure 2**.** Probabilities of belonging to physical disability trajectories (Expanded Disability Status Scale) conditioned on belonging to each fatigue trajectory (Fatigue Scale for Motor and Cognitive function), quantifying the connection between fatigue and physical disability in relapsing-remitting multiple sclerosis people over 11 years following disease-modifying therapy switch.

**
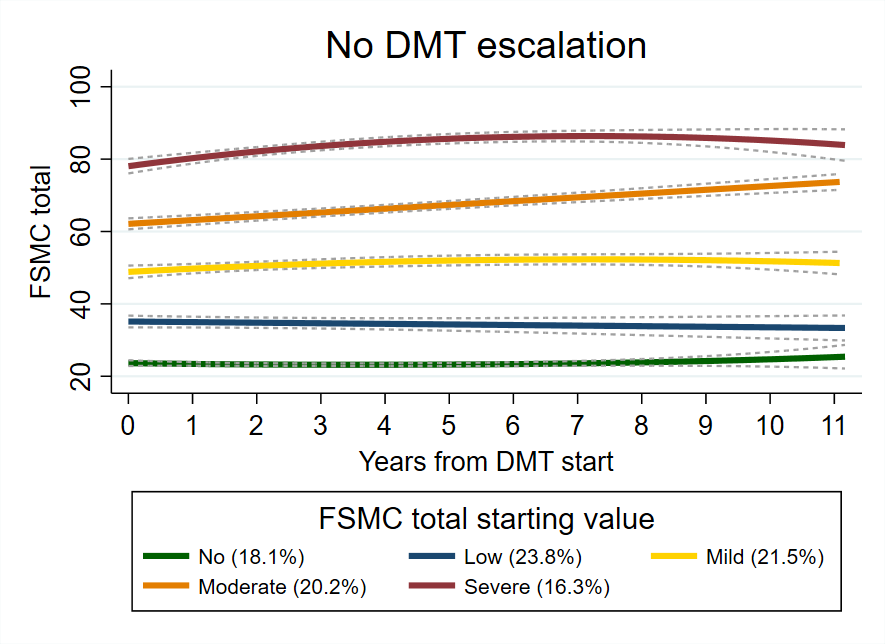
**

eFigure 3. Trajectories of FSMC total over years from DMT start of study participants of the first DMT cohort who did not switch to a more effective DMT.

**
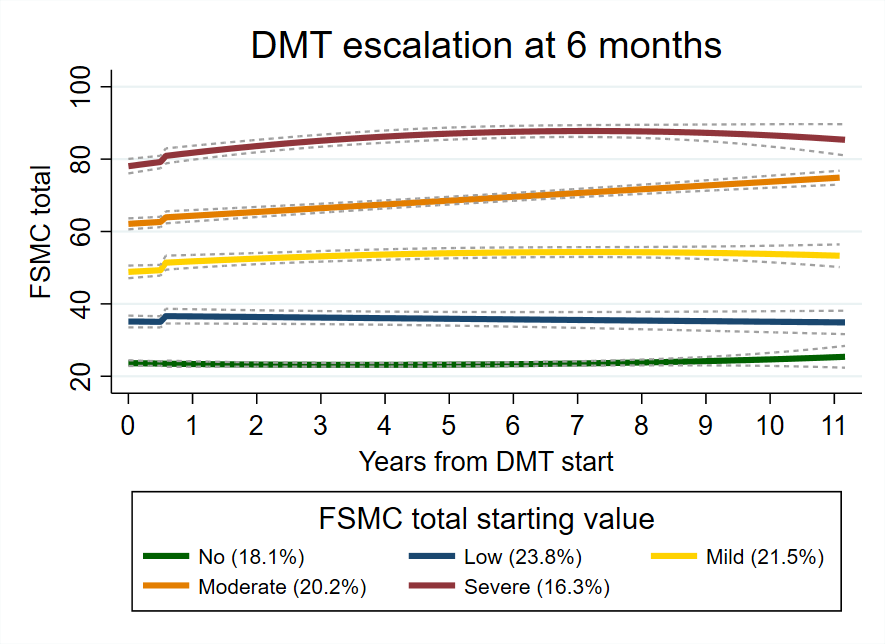
**

eFigure 4. Trajectories of FSMC total over years from DMT start of study participants of the first DMT cohort who switched to a more effective DMT 6 months after first line DMT start.

**
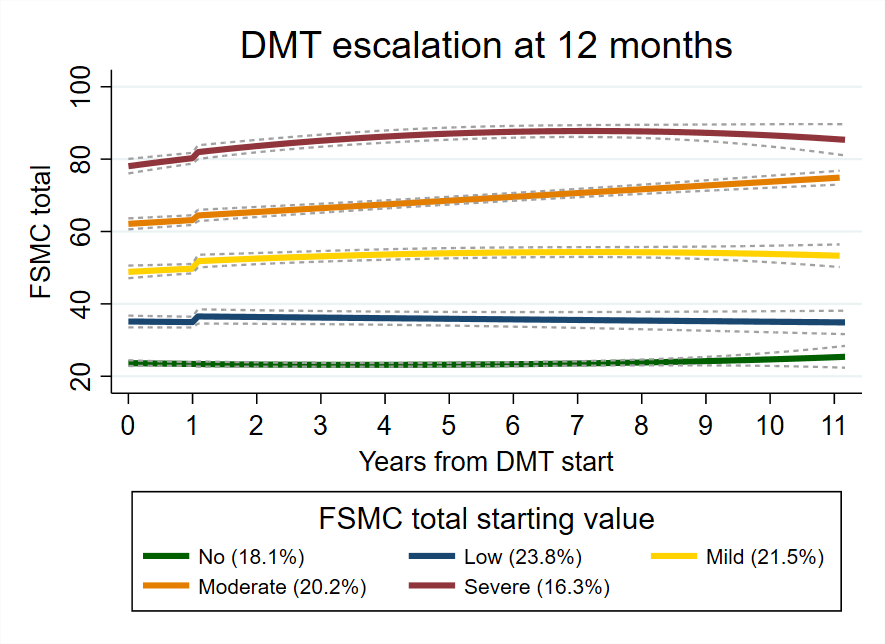
**

eFigure 5**.** Trajectories of FSMC total over years from DMT start of study participants of the first DMT cohort who switched to a more effective DMT 12 months after first line DMT start.

**
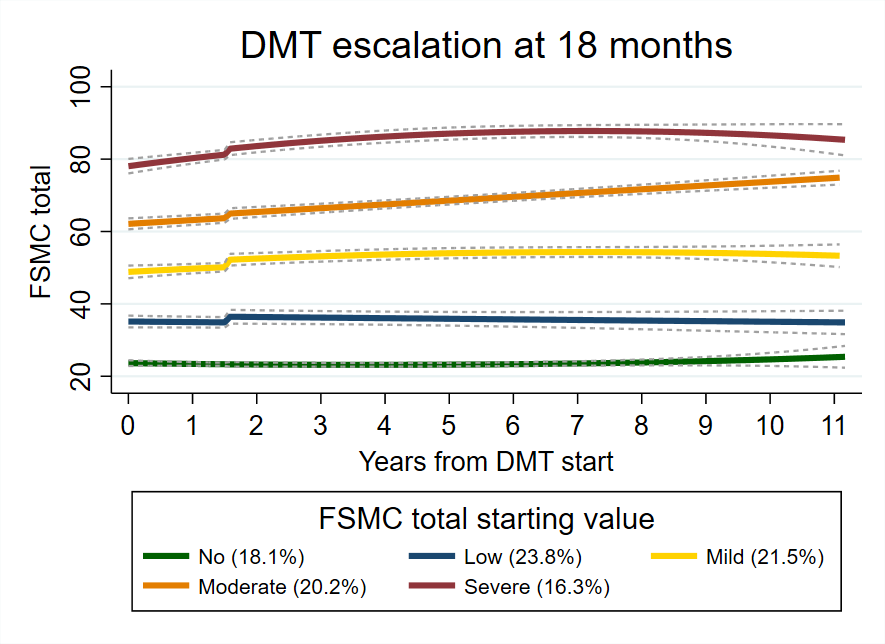
**

eFigure 6. Trajectories of FSMC total over years from DMT start of study participants of the first DMT cohort who switched to a more effective DMT 18 months after first line DMT start.

**
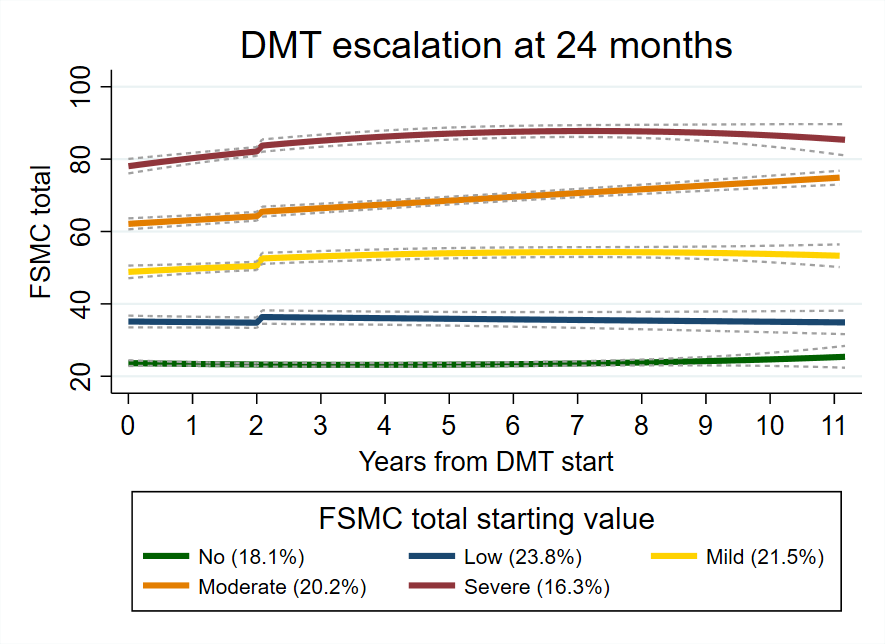
**

eFigure 7**.** Trajectories of FSMC total over years from DMT start of study participants of the first DMT cohort who switched to a more effective DMT 24 months after first line DMT start.

**
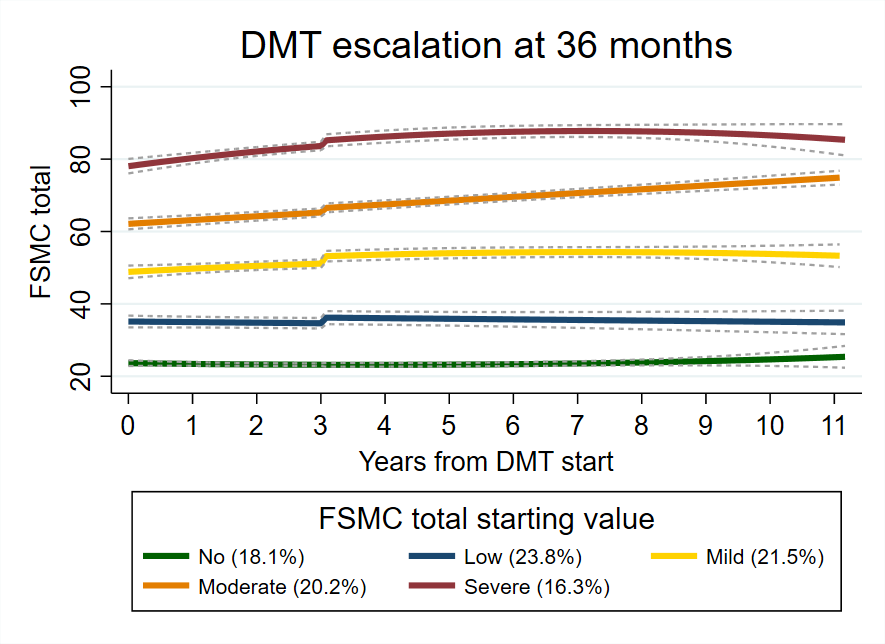
**

eFigure 8. Trajectories of FSMC total over years from DMT start of study participants of the first DMT cohort who switched to a more effective DMT 36 months after first line DMT start.

**
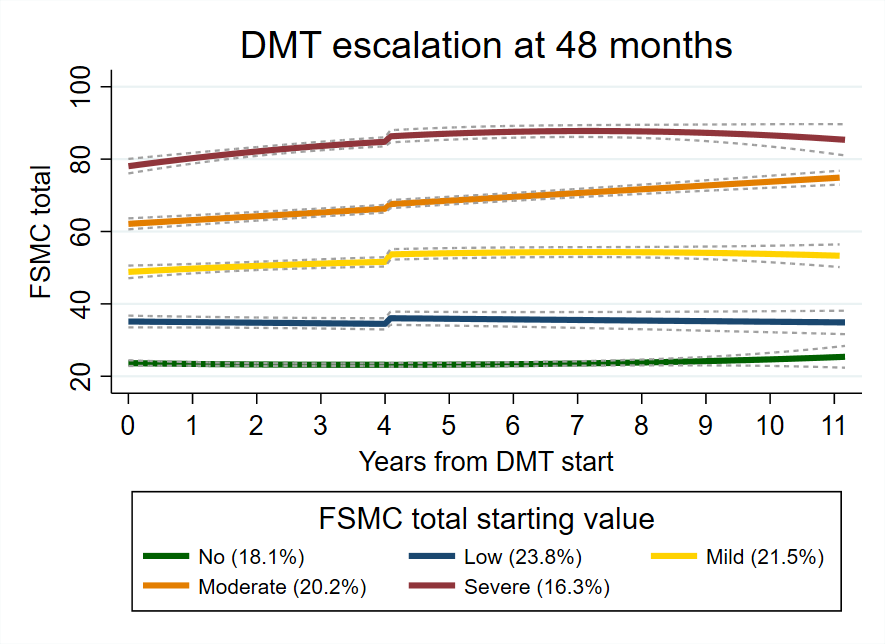
**

eFigure 9. Trajectories of FSMC total over years from DMT start of study participants of the first DMT cohort who switched to a more effective DMT 48 months after first line DMT start.

**
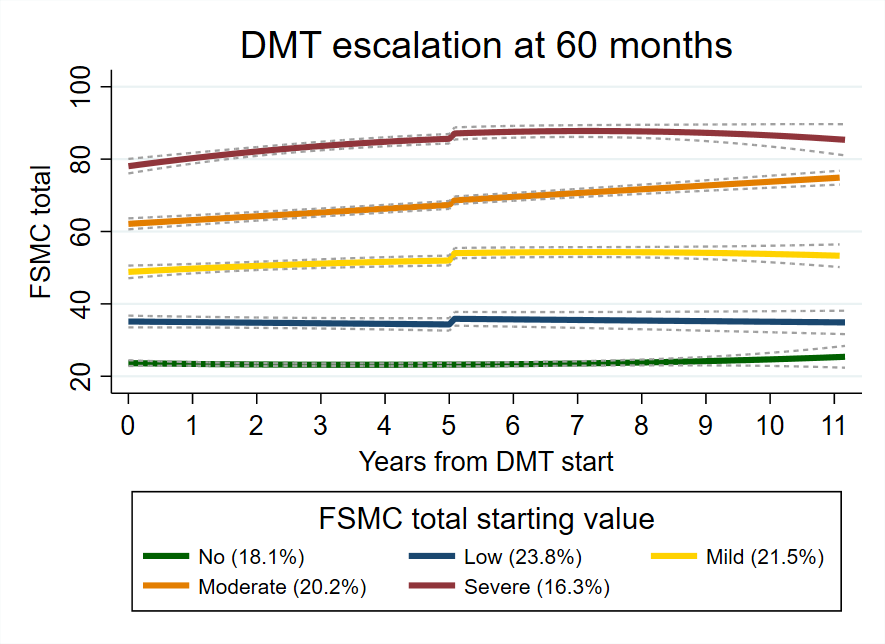
**

eFigure 10. Trajectories of FSMC total over years from DMT start of study participants of the first DMT cohort who switched to a more effective DMT 60 months after first line DMT start.

**
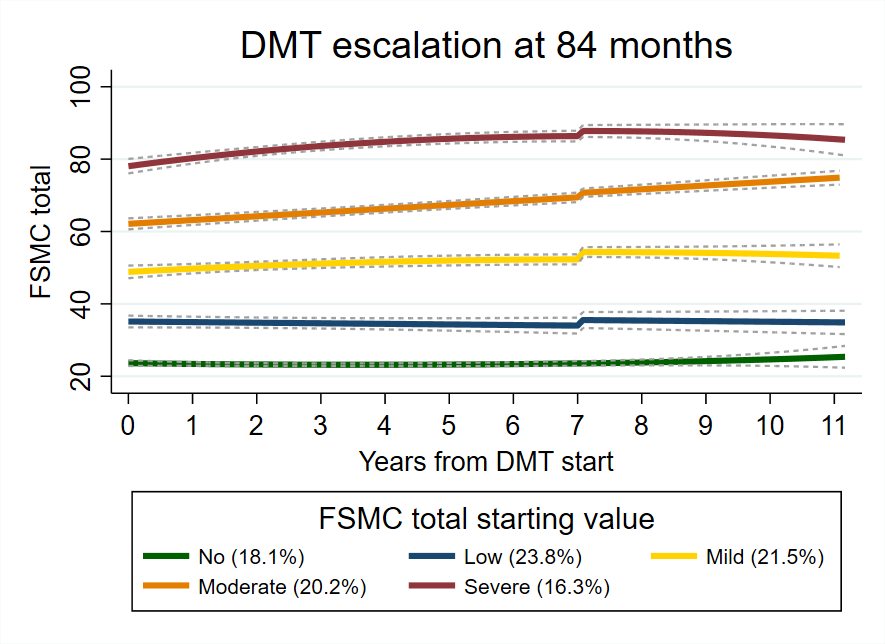
**

eFigure 11. Trajectories of FSMC total over years from DMT start of study participants of the first DMT cohort who switched to a more effective DMT 84 months after first line DMT start.

**
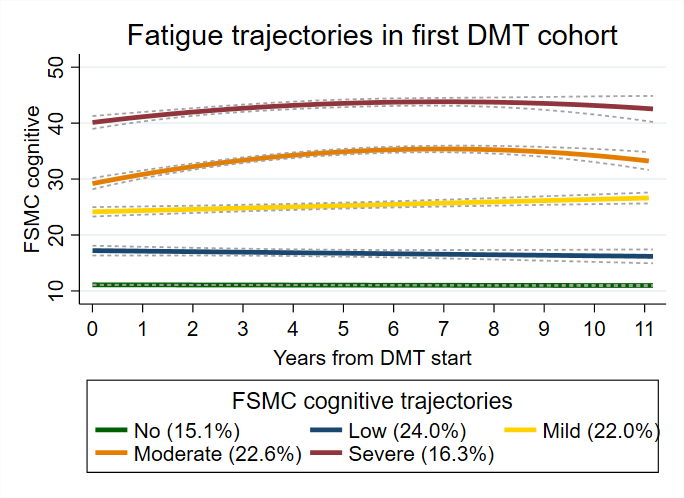
**

**
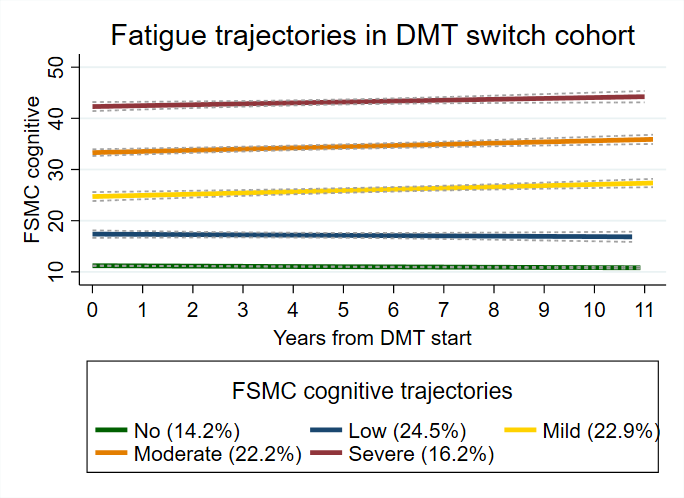
**

eFigure 12. Trajectories of the Fatigue Scale for Motor and Cognitive function (FSMC) cognitive and corresponding 95% Cis (dotted lines), over 11 years following first (upper panel) and switch (lower panel) disease-modifying therapy (DMT) initiation. Trajectory group legends describe the proportion of participants in each group relative to the entire study population.

**
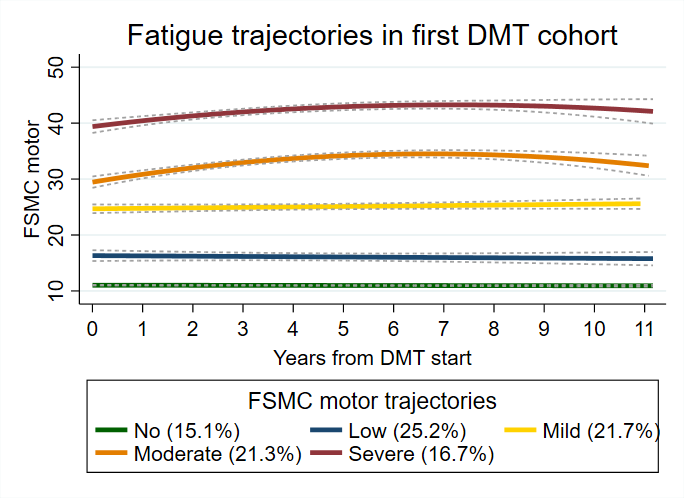
**

**
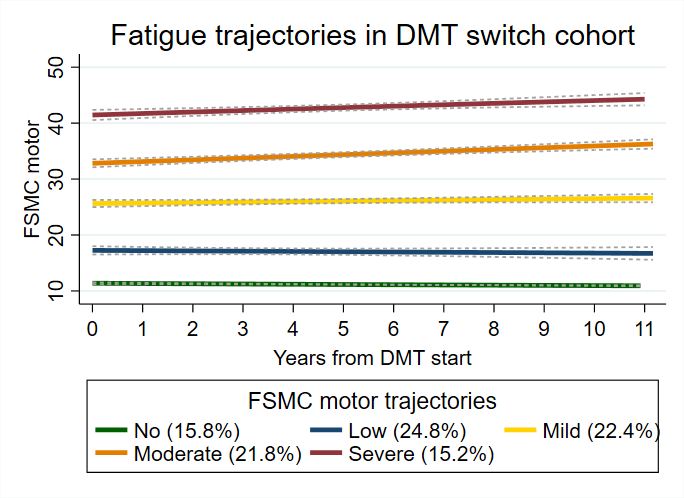
**

eFigure 13. Trajectories of the Fatigue Scale for Motor and Cognitive function (FSMC) motor and corresponding 95% Cis (dotted lines), over 11 years following first (upper panel) and switch (lower panel) disease-modifying therapy (DMT) initiation. Trajectory group legends describe the proportion of participants in each group relative to the entire study population.

## References for supplementary material

1. Ludvigsson JF, Appelros P, Askling J, et al. Adaptation of the Charlson Comorbidity Index for Register-Based Research in Sweden. *Clin Epidemiol*. 2021;13:21-41. doi:10.2147/clep.S282475

2. Sharrack B, Hughes RA. The Guy's Neurological Disability Scale (GNDS): a new disability measure for multiple sclerosis. *Mult Scler*. 1999;5(4):223-33. doi:10.1177/135245859900500406
